# Genomic and Metabolomic Investigation of a Rhizosphere Isolate *Streptomyces netropsis* WLXQSS-4 Associated with a Traditional Chinese Medicine

**DOI:** 10.3390/molecules26082147

**Published:** 2021-04-08

**Authors:** Songya Zhang, Lingxiao Zhang, Jing Zhu, Hong Chen, Zhicong Chen, Tong Si, Tao Liu

**Affiliations:** 1 Shenzhen Institute of Synthetic Biology, Shenzhen Institutes of Advanced Technology, Chinese Academy of Sciences, Shenzhen 518055, China; sy.zhang2@siat.ac.cn (S.Z.); jing.zhu@siat.ac.cn (J.Z.); zc.chen@siat.ac.cn (Z.C.); 2 Department of Natural Products Chemistry, School of Pharmacy, China Medical University, Shenyang 110122, China; 13022551727@163.com (L.Z.); chenhong950914@163.com (H.C.)

**Keywords:** rhizospheric microorganism, *Streptomyces*, traditional Chinese medicine, genome, secondary metabolites, biological activities

## Abstract

Rhizosphere microorganisms play important ecological roles in promoting herb growth and producing abundant secondary metabolites. Studies on the rhizosphere microbes of traditional Chinese medicines (TCMs) are limited, especially on the genomic and metabolic levels. In this study, we reported the isolation and characterization of a *Steptomyces netropsis* WLXQSS-4 strain from the rhizospheric soil of *Clematis manshurica* Rupr. Genomic sequencing revealed an impressive total of 40 predicted biosynthetic gene clusters (BGCs), whereas metabolomic profiling revealed 13 secondary metabolites under current laboratory conditions. Particularly, medium screening activated the production of alloaureothin, whereas brominated and chlorinated pimprinine derivatives were identified through precursor-directed feeding. Moreover, antiproliferative activities against Hela and A549 cancer cell lines were observed for five compounds, of which two also elicited potent growth inhibition in *Enterococcus faecalis* and *Staphylococcus aureus*, respectively. Our results demonstrated the robust secondary metabolism of *S. netropsis* WLXQSS-4, which may serve as a biocontrol agent upon further investigation.

## 1. Introduction

The plant-associated microbial communities in the rhizosphere sustain plant growth and health in many manners, including seed germination, nitrogen fixation, stress robustness, and pathogen defense. Among all rhizosphere microorganisms, actinomycetes not only constitute a substantial portion of the soil microbial community but also exhibited unique characteristics [1]. For example, actinomycetes maintain close physical contact with soil particles and plants through filaments and sporulation. They also produce abundant secondary metabolites with antifungal, insecticidal, and antibacterial activities, which assist plants in fighting pathogens. Moreover, actinomycetes produce phytohormones that solubilize phosphate, which is an essential nutrition to support plant growth [2].

Rhizosphere microorganisms exert substantial impacts on the agriculture and medicinal properties of plants used in traditional Chinese medicines (TCMs). Alternation of soil microbial communities is an important reason why many cultivated TCMs cannot be cropped continuously [3]. Additionally, the bioactive secondary metabolites in some TCMs exhibited distinct correlations with plant-associated microbes [4]. For example, some rhizosphere microbes may serve as environmental stimuli to promote biosynthesis of an active TCM component. Despite the importance of rhizosphere microorganisms, they remain largely uncharacterized mainly due to the challenges in cultivation under laboratory conditions. We and others recently carried out a series of studies on the microorganisms derived from the rhizosphere of Chinese herbal medicinal plants [4,5,6], including *Clematis manshurica* Rupr., *Polygonatum odoratum* (Mill.) Druce, and *Dictamnus dasycarpus* Turcz.

In this study, a plant growth-promoting *Streptomyces* strain, dubbed *S. netropsis* WLXQSS-4, was isolated from the rhizosphere of *Clematis manshurica* Rupr. We performed genome sequencing and annotation, which predicted a rich collection of biosynthetic gene clusters (BGCs). Metabolomic analyses revealed the production of a series of secondary metabolites, such as tryptophan derivatives including a new optical isomer (compound **3**). For some derivatives, antagonistic activities were observed against several pathogenic bacteria. Notably, *Clematis manshurica* Rupr. produces many indole alkaloids [7], and it is possible that *S. netropsis* WLXQSS-4 may contribute indole precursors to its host production of tryptophan-derived molecules. Moreover, the cryptic gene cluster of alloaureothin was activated through the one strain–many compounds (OSMAC) strategy. One new brominated and three chlorinated pimprinine derivatives were also identified through the precursor-directed feeding method. 

## 2. Results

### 2.1. General Characterization of S. netropsis WLXQSS-4

**Figure 1:**
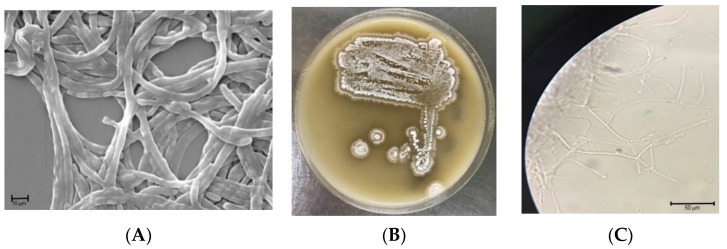
Phenotype characterization of S. netropsis WLXQSS-4. (**A**) Scanning electron microscope (SEM) (magnification of 5000) analysis; (**B**) colony morphological characteristics; (**C**) and optical microscopic image of *S. netropsis* WLXQSS-4. The pictures were taken after 48 h cultivation at 30 °C.

The isolate *S. netropsis* WLXQSS-4 produced white fuzzy, small to medium-sized colonies, with pigmentation not uniformly expressed across colonies. The strain produced whitish, fuzzy colonies when cultured on MS agar plates. The spores had a smooth surface, were oblong in shape, and were arranged in chains when examined by Scanning Electron Microscopic (SEM) analysis (Figure 1).

**Figure 2:**
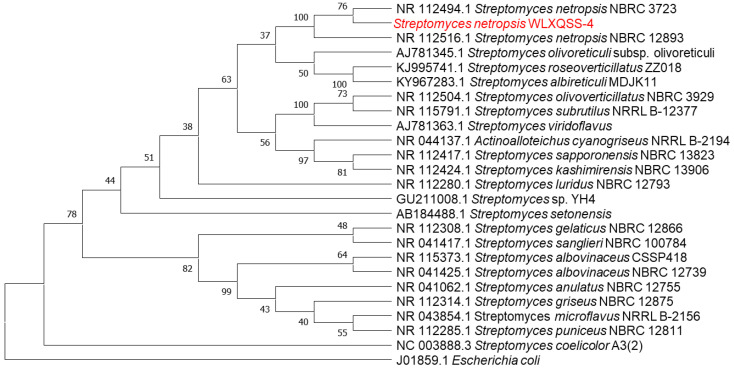
Phylogenetic analysis of 16S rRNA gene sequences of the *S. netropsis* WLXQSS-4 isolated from rhizosphere. Sequences were aligned through ClustalW using MEGA 7 software. Phylogenetic tree was constructed using Maximum Likelihood method. Bootstrap values are shown as percentages of 1000 replicates.

Molecular analysis indicated that the isolated strain belongs to the genus *Streptomyces*. In particular, an unsupervised nucleotide BLAST analysis based on the 16S rRNA gene from *S. netropsis* WLXQSS-4 with the 16S rRNA gene from different *Streptomyces* was performed to determine their phylogenetic relationships. *Escherichia coli* (J01859.1) was chosen as an out-group. Within Streptomyces species, this isolate keeps its closest kinship with Streptomyces *S. netropsis* NBRC 3723 and *S. netropsis* NBRC 12,893 with 100% identity (Figure 2). The 16S rRNA gene sequence of *S. netropsis* WLXQSS-4 was submitted to the National Microbiology Data Center (NMDC) under accession number NMDCN000022U.

### 2.2. Genome Sequencing and Annotation of S. netropsis WLXQSS-4

To understand the biosynthetic potential of *S. netropsis* WLXQSS-4, its genome was sequenced using a combination of Illumina HiSeq 4000 and PacBio sequencing technology. The assembled genome of *S. netropsis* WLXQSS-4 is 8,324,019 bp in length and consists of a linear chromosome with a mean GC content of 71.32% (Figure S1). The genome contains 7115 protein-coding sequences, 21 rRNA genes, and 78 tRNA genes identified through bioinformatics analysis. The genome sequence of *S. netropsis* WLXQSS-4 has been deposited in the NMDC database with the accession number of NMDC60014575. Table 1 provides the overview of the genomic features of this strain.

Clusters of Orthologous Groups of proteins (COGs) from the chromosome of the strain *S. netropsis* WLXQSS-4 were annotated by the COG database [8]. The COG annotation results show that 6702 genes have at least one biological function annotation (Figure S2).

The genome sequence was subjected to bioinformatics analysis for secondary metabolism, and antiSMASH and Prism software predicted 40 BGCs, which potentially encoding nonribosomal peptides (NRPs), polyketides (PKs), ribosomally synthesized and post-translationally modified peptides (RiPPs), terpenes, siderophores, and other types of metabolites (Table S1).

### 2.3. Metabolome Investigation on Strain S. netropsis WLXQSS-4

#### 2.3.1. Analysis of the Secondary Metabolites of *S. netropsis* WLXQSS-4

Motivated by the noteworthy antibacterial activity of the *S. netropsis* WLXQSS-4 colony during initial bioactivity assay, the secondary metabolic profile of strain *S. netropsis* WLXQSS-4 was analyzed by high-resolution mass spectrometry (HR-MS), and our results showed that this strain can produce significant abundance of secondary metabolites. Using mass spectrometry (MS), the crude extracts were subjected to the serial chromatography method to yield purified compounds. The chemical structures of purified compounds were determined based on NMR and MS (Figure 3 and Supplementary Figures S3–S32).

Interestingly, a group of tryptophan-derived natural products was discovered from the extracts, including a series of pimprinine derivatives, indole, and flazin. In light of the high-frequency appearance of tryptophan derivatives, we investigated whether *S. netropsis* WLXQSS-4 may metabolize halogenated tryptophan congeners. A precursor-direct feeding experiment was performed in A medium supplemented with H-Trp(6-Br)-OH (CAS No: 52448-17-6) and H-Trp(6-Cl)-OH (CAS No: 33468-35-8). The resultant crude extracts were subjected to HR-MS analyses, and the raw data were converted and submitted into Global Natural Products Social Molecular Networking (GNPS) [9] for MS/MS-based molecular networking analysis.

Based on the similarity of MS/MS fragmentation patterns, a network of secondary metabolites was created (Figure 4), including a sub-cluster for pimprinine derivatives. Three chlorinated pimprinine congeners (*m*/*z* [M − H]^−^ 231.0328, 261.0436, and 245.0485) and one brominated congener (*m*/*z* [M − H]^−^ 288.9984) were identified (Figures S33–S36). Those new derivatives were depicted in Figure 4. Based on our results and the previous literature [10,11], we postulated a possible BGC for pimprinine (Figure S37). Unfortunately, gene inactivation experiments in *S. netropsis* WLXQSS-4 have been so far unsuccessful in confirming our hypothesis.

The biosynthesis machinery of flazin in *S. netropsis* WLXQSS-4 is not yet clear (Figure S38). One possible mechanism of l-tryptophan and oxaloacetaldehyde cyclization is mediated by McbB-like Pictet-Spengler cyclase [12]. To examine this possibility, we obtained the complete genome sequence of *S. netropsis* WLXQSS-4, within which we searched for homologous protein sequences of McbA, McbB, CyaB, and McbC [12]. Unfortunately, no McbB homologs were identified in this strain. Therefore, the biosynthesis of flazin may undertake an unknown mechanism. An alternative biosynthetic pathway was proposed to integrate tryptophan with hydroxymethylfurfural (Figure S38).

#### 2.3.2. Activation of a Cryptic Gene Cluster of *S. netropsis* WLXQSS-4 by Medium Screening

**Figure 3:**
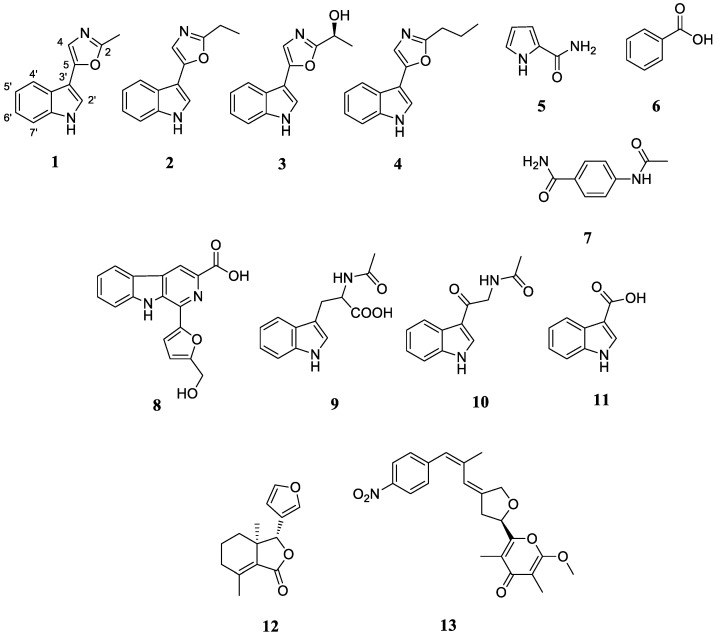
Secondary metabolites isolated from the crude extracts of *S. netropsis* WLXQSS-4.

**Figure 4:**
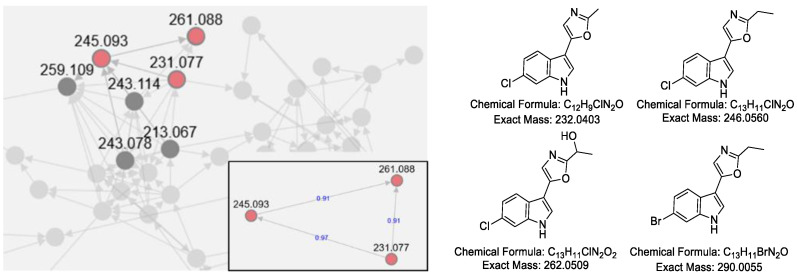
Molecular network and predicted chemical structures of the halogenated pimprinine derivatives produced during precursor-direct feeding. The network was generated by GNPS. Each node represents *m*/*z* value of the parent ion, and the edges are labeled by cosine score in the zoomed figure.

**Figure 5:**
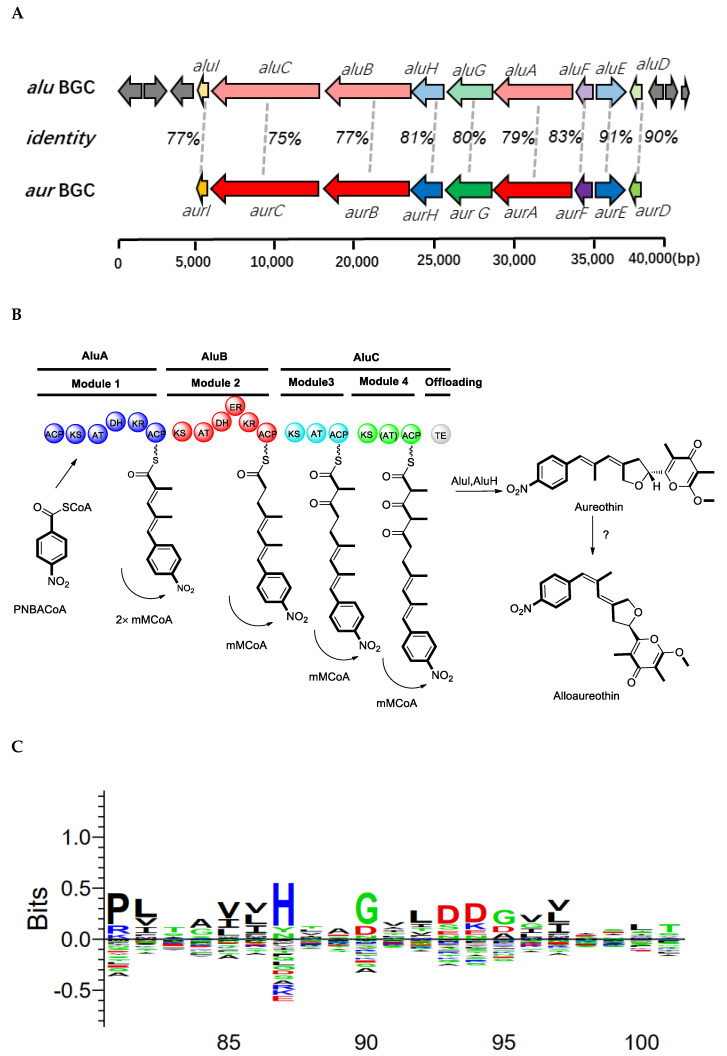
Biosynthetic gene clusters and proposed pathway for alloaureothin biosynthesis in *S. netropsis* WLXQSS-4. (**A**) Gene organization of the *alu* and *aur* clusters, the deduced functions of the genes are labeled in color and summarized in Table S2. (**B**) Proposed biosynthesis of *alu* in *S. netropsis* WLXQSS-4. Abbreviation notation: ACP, acyl carrier protein; C, condensation; A, adenylation; KR, ketoreductase; KS, ketosynthase; TE, thioesterase; mmCoA, methylmalonyl-CoA; mCoA, malonyl-CoA. (**C**) A sequence logo showing the presence of the conserved “LDD” motif in AluA using the sequences alignment with other 107 KR domains (diagram created by Seq2Logo).

The one strain–many compounds (OSMAC) strategy has been demonstrated as a facile and powerful tool for activating cryptic BGCs in microorganisms [13]. Based on this approach, we observed a new peak when *S. netropsis* WLXQSS-4 was cultivated in the NL-19 medium. Through serial purification, HR-MS and NMR experiments (Supplementary Figures S39–S42), this compound was identified as compound **13** (alloaureothin) (Figure 3 and Figure 5) via comparison with spectral data in the literature [14].

The biosynthetic mechanism of aureothin has been reported [15]. Here, we proposed a possible BGC of alloaureothin by bioinformatic analysis of the *S. netropsis* WLXQSS-4 genome. The proposed ~40 kb BGC is at the sub-telomeric region and consists of 15 open reading frames. Nine proteins were assigned possible functions based on their homology to proteins of known function (Table S2). This BGC is highly similar in both gene sequences and cluster organization to the previously published BGC of aureothin, suggesting a similar biosynthetic mechanism (Figure 5). Therefore, an assembly logic of alloaureothin was proposed (Figure 5). The core part of the alloaureothin gene cluster contains three genes encoding polyketide synthetase (*aluA*, *aluB*, *aluC*) responsible for the PKS molecular backbone assembly (Figure 5A,B). Among them, AluA architecture is composed of the KS, AT, DH, KR, and ACP domains, and it executes two times iteratively elongation steps of the unsaturated chain. The polyketide backbone is further modified by several tailoring enzymes, AluF as an N-oxygenase is responsible for the generation of the nitro aryl moiety, gene *aluH* encodes a cytochrome P450 monooxygenase, which is predicted for the stereoselective synthesis of the furan ring. Other genes putatively are recognized to be relevant with the synthesis of starter unit *p*-nitrobenzoate (PNBA) (*aluE and aluG*), pyrone methylation (*aluI*), and pathway regulation (*aluD*).

The configuration of the alloaureothin diene was assigned as *E* and *Z*, which was different from aureothin containing a diene scaffold with *E*, *E* configuration. This single stereochemistry change results in a significant difference in physico-chemical properties [14]. Similar to the *aur* BGC system, AluA iteratively executes the assembly of two malonylmethyl groups, but interestingly, it generates the diene with *trans-* and *cis-*, which are two different configurations. One possible origin for the *cis* double bond is through the action of an A-type KR to produce an alcohol with the opposite stereochemical configuration (L-3-hydroxyacyl intermediate). However, the sequence alignment exhibits that the KR from AluA has the characteristic motifs “LDD” in their active site (Figure 5C); therefore, it was designated as a reductase owing B-type stereochemistry [16,17]. Thus, an alternative route was proposed, the *cis*-alkene moiety may undergo an isomerization of the *trans* stereochemistry, which may be catalyzed by a post-tailoring enzyme as shown in the biosynthesis of phoslactomycin [18]. The biosynthetic mechanism of alloaureothin demonstrates the plasticity of polyketide backbone assembly.

#### 2.3.3. Structural Elucidation

Compound **1** (pimprinine): yellow oil; (+)-ESI-MS *m*/*z* 199.1 [M + H]^+^; (−)-ESI-MS *m*/*z* 197.0 [M − H]^−^; ^1^H-NMR (600 MHz, CD_3_OD) δ: 2.52 (3H, s, 2-Me), 7.14 (1H, s, H-4), 7.15 (1H, m, H-5′), 7.20 (1H, m, H-6′), 7.43 (1H, d, *J* = 8.1 Hz, H-7′), 7.59 (1H, s, H-2′), 7.78 (1H, d, *J* = 8.1 Hz, H-4′); ^13^C-NMR (150 MHz, CD_3_OD) δ: 13.5 (2-Me), 105.5 (C-3′), 112.8 (C-7′), 119.2 (C-4), 120.4 (C-4′), 121.3 (C-5′), 123.4 (C-6′), 123.7 (C-2′), 125.3 (C-3′a), 138.2 (C-7′a), 150.2 (C-5), 160.8 (C-2) [19,20].

Compound **2** (pimprinethine): colorless needles (MeOH); ^1^H-NMR (600 MHz, DMSO-*d*_6_) δ: 1.30 (3H, t, *J* = 7.6 Hz, H-7), 2.81 (2H, q, *J* = 7.5 Hz, H-6), 7.13 (1H, t, *J* = 7.5 Hz, H-5′), 7.19 (1H, t, *J* = 7.5 Hz, H-6′), 7.27 (1H, s, H-4), 7.45 (1H, d, *J* = 8.1 Hz, H-7′), 7.72 (1H, d, *J* = 2.5 Hz, H-2′), 7.82 (1H, d, *J* = 8.1 Hz, H-4′), 11.51 (1H, brs, 1′-NH); ^13^C-NMR (150 MHz, DMSO-*d*_6_) δ: 11.2 (C-7), 21.0 (C-6), 103.9 (C-3′), 112.0 (C-7′), 119.0 (C-4), 119.5 (C-4′), 120.0 (C-5′), 122.1 (C-6′), 122.9 (C-2′), 123.5 (C-3′a), 136.3 (C-7′a), 147.2 (C-5), 162.5 (C-2) [21,22].

Compound **3** ((−)-pimprinol A): yellow oil; [*α*]_D_^25^ = −1.9° (*c* 0.1, MeOH); (+)-ESI-MS *m*/*z* 229.2 [M + H]^+^, *m*/*z* 251.2 [M + Na]^+^; (-)-ESI-MS *m*/*z* 227.0 [M − H]^−^; ^1^H-NMR (600 MHz, CD_3_OD) δ: 1.63 (3H, d, *J* = 6.7 Hz, H-7), 4.96 (1H, q, *J* = 6.7 Hz, H-6), 7.16 (1H, t, *J* = 7.4 Hz, H-5′), 7.21 (1H, t, *J* = 7.4 Hz, H-6′), 7.22 (1H, s, H-4), 7.44 (1H, d, *J* = 8.1 Hz, H-7′), 7.65 (1H, s, H-2′), 7.83 (1H, d, *J* = 8.1 Hz, H-4′); ^13^C-NMR (150 MHz, CD_3_OD) δ: 21.5 (C-7), 64.2 (C-6), 105.4 (C-3′), 112.8 (C-7′), 119.2 (C-4), 120.5 (C-4′), 121.4 (C-5′), 123.5 (C-6′), 124.0 (C-2′), 125.4 (C-3′a), 138.2 (C-7′a), 150.5 (C-5) [20].

Compound **4** (WS-30581 A): white solid; ^1^H-NMR (600 MHz, DMSO-*d*_6_) δ: 0.98 (3H, t, *J* = 7.3 Hz, H-8), 1.77 (2H, m, H-7), 2.77 (2H, t, *J* = 7.3 Hz, H-6), 7.13 (1H, t, *J* = 7.4 Hz, H-5′), 7.19 (1H, t, *J* = 7.4 Hz, H-6′), 7.27 (1H, s, H-4), 7.45 (1H, d, *J* = 8.1 Hz, H-7′), 7.71 (1H, d, *J* = 2.5 Hz, H-2′), 7.81 (1H, d, *J* = 8.1 Hz, H-4′), 11.51 (1H, brs, 1′-NH). ^13^C-NMR (150 MHz, DMSO-*d*_6_) δ: 13.5 (C-8), 20.1 (C-7), 29.3 (C-6), 112.0 (C-7′), 119.1 (C-4), 120.0 (C-5′), 122.9 (C-2′), 136.3 (C-7′a) [21,23].

Compound **5** (pyrrole-2-carboxamide): white solid; ^1^H-NMR (600 MHz, DMSO-*d*_6_) δ: 6.05 (1H, m, 4-H), 6.75 (1H, m, 5-H), 6.83 (1H, m, 3-H), 6.88 (1H, brs, 1′-NH_2_), 7.45 (1H, brs, 1′-NH_2_), 11.37 (1H, brs, 1-NH); ^13^C-NMR (150 MHz, DMSO-*d*_6_) δ: 108.5 (C-4), 110.6 (C-3), 121.3 (C-5), 126.3 (C-2), 162.3 (2-CO) [24].

Compound **6** (benzoic acid): white solid; ^1^H-NMR (600 MHz, CDCl_3_) δ: 7.48 (2H, m, H-3, H-5), 7.62 (1H, t, *J* = 7.1 Hz, H-4), 8.11 (2H, d, *J* = 7.9Hz, H-2, H-6); ^13^C-NMR (150 MHz, CDCl_3_) δ: 128.7 (C-3, C-5), 129.3 (C-1), 130.4 (C-2, C-6), 133.9 (C-4), 170.7 (C-1′) [25].

Compound **7** (4-acetamidobenzamide): white solid; (+)-HRESI-MS *m*/*z* 179.0809 [M + H]^+^ (calcd. for C_9_H_11_N_2_O_2_ 179.0815); ^1^H-NMR (600 MHz, DMSO-*d*_6_) δ: 2.06 (3H, s, H-9), 7.21 (1H, brs, 7-NH_2_), 7.62 (2H, d, *J* = 8.4 Hz, H-3, H-5), 7.80 (2H, d, *J* = 8.4 Hz, H-2, H-6), 7.83 (1H, brs, 7-NH_2_), 10.14 (1H, s, 4-NH); ^13^C-NMR (150 MHz, DMSO-*d*_6_) δ: 24.1 (C-9), 118.0 (C-3, C-5), 128.3 (C-2, C-6), 128.5 (C-1), 141.9 (C-4), 167.4 (C-7), 168.7 (C-8) [26,27].

Compound **8** (flazin): yellow oil; ^1^H-NMR (600 MHz, DMSO-*d*_6_) δ: 4.68 (2H, s, 2′-CH_2_-), 5.50 (1H, brs, H-COOH), 6.62 (1H, d, *J* = 3.1 Hz, H-3′), 7.35 (1H, t, *J* = 7.5 Hz, H-6), 7.43 (1H, d, *J* = 3.1 Hz, H-4′), 7.65 (1H, t, *J* = 7.5 Hz, H-7), 7.83 (1H, d, *J* = 8.2 Hz, H-8), 8.42 (1H, d, *J* = 8.2 Hz, H-5), 8.84 (1H, s, H-4), 11.60 (1H, s, 9-NH), 12.68 (1H, brs, 2′-OH); ^13^C-NMR (150 MHz, DMSO-*d*_6_) δ: 55.9 (2′-CH_2_-), 109.2 (C-3′), 111.1 (C-4′), 112.8 (C-8), 115.7 (C-4), 120.5 (C-6), 121.0 (C-4a, 5a), 122.0 (C-5), 128.9 (C-7), 129.8 (C-3), 131.9 (C-9a), 132.5 (C-1), 141.4 (C-8a), 151.2 (C-5′), 157.3 (C-2′), 166.4 (3-COOH) [28].

Compound **9** (*N*-acetyltryptophan): colorless needles (MeOH); [*α*]_D_^25^ = 16.5° (*c* 0.1, H_2_O); (−)-ESI-MS *m*/*z* 245.12 [M − H]^−^; H-NMR (600 MHz, DMSO-*d*_6_) δ: 1.75 (3H, s, 4′-Me), 2.95 (1H, dd, *J* = 7.1 Hz, 13.9 Hz, H-2′), 3.20 (2H, m, H-1′), 6.92 (1H, t, *J* = 7.1 Hz, H-5), 7.01 (1H, t, *J* = 7.1 Hz, H-6), 7.08 (1H, s, H-2), 7.28 (1H, d, *J* = 7.7 Hz, H-4), 7.49 (1H, d, *J* = 7.7 Hz, H-7), 10.69 (1H, s, 3′-NH); ^13^C-NMR (150 MHz, DMSO-*d*_6_) δ: 23.0 (CH_3_), 27.6 (C-1′), 54.8 (C-2′), 109.5 (C-3), 111.1 (C-7), 118.0 (C-5), 118.5 (C-6), 120.5 (C-4), 123.2 (C-2), 128.0 (C-3a), 135.9 (C-7a) [29].

Compound **10** (*N*-acetyl-β-oxotryptamine): white solid; (+)-ESI-MS *m*/*z* 239.12 [M + Na]^+^; ^1^H-NMR (600 MHz, CD_3_OD) δ: 2.08 (3H, s, 5′-Me), 4.60 (2H, s, H-2′), 7.23 (2H, m, H-5, H-6), 7.46 (1H, d, *J* = 7.6 Hz, H-7), 8.22 (1H, d, *J* = 7.4 Hz, H-4), 8.23 (1H, s, H-2); ^13^C-NMR (150 MHz, CD_3_OD) δ: 22.5 (C-5′), 47.1 (C-2′), 113.0 (C-7), 115.8 (C-3), 122.7 (C-4), 123.3 (C-5), 124.4 (C-6), 126.9 (C-3a), 134.3 (C-2), 138.3 (C-7a), 173.5 (C-4′), 192.0 (C-1′) [30].

Compound **11** (1*H*-indole-3-carboxylic acid): yellowish solid; ^1^H-NMR (600 MHz, CD_3_OD) δ: 7.18 (2H, m, H-5, H-6), 7.44 (1H, d, *J* = 7.6 Hz, H-7), 7.94 (1H, s, H-2), 8.06 (1H, d, *J* = 7.3 Hz, H-4); ^13^C-NMR (150 MHz, CD_3_OD) δ: 108.7 (C-3), 112.9 (C-7), 122.0 (C-4), 122.4 (C-5), 123.6 (C-6), 127.6 (C-3a), 133.4 (C-2), 138.2 (C-7a), 169.2 (3-COOH) [31].

Compound **12** (fraxinellone): colorless needles (CDCl_3_); (+)-HRESI-MS *m*/*z* 233.1173 [M + H]^+^ (calcd. for C_14_H_17_O_3_ 233.1172); ^1^H-NMR (600 MHz, CDCl_3_) δ: 0.86 (3H, s, 3a-Me), 1.45 (1H, m, H-4), 1.73 (1H, m, H-4), 1.82 (2H, m, H-5), 2.13 (3H, s, 7-Me), 2.18 (1H, dd, *J* = 7.3 Hz, 10.8 Hz, H-6), 2.28 (1H, dd, *J* = 6.5 Hz, 19.7 Hz, H-6), 4.88 (1H, s, H-3), 6.35 (1H, d, *J* = 1.0 Hz, H-4′), 7.44 (1H, t, *J* = 1.7 Hz, H-5′), 7.47 (1H, s, H-2′); ^13^C-NMR (150 MHz, CDCl_3_) δ: 18.4 (C-5), 18.6 (7-Me), 20.5 (3a-Me), 31.8 (C-6), 32.2 (C-4), 43.1 (C-3a), 83.6 (C-3), 108.7 (C-4′), 120.8 (C-3′), 127.6 (C-7), 139.9 (C-2′), 143.6 (C-5′), 148.7 (C-7a), 170.0 (C-1) [32,33].

Compound **13** (alloaureothin): yellow oil; [*α*]_D_^25^ = −14.8° (*c* 0.12, CHCl_3_); (+)-ESI-MS *m*/*z* 398.27 [M + H]^+^, *m*/*z* 420.24 [M + Na]^+^; (−)-HRESI-MS *m*/*z* 396.1455 [M − H]^−^ (calcd. for C_22_H_22_NO_6_ 396.1453); ^1^H-NMR (600 MHz, CDCl_3_) δ: 1.86 (3H, s, 2-Me), 2.01 (3H, s, 4-Me), 2.07 (3H, d, *J* = 1.1 Hz, 11-Me), 2.89 (1H, dd, *J* = 7.4 Hz, 15.4 Hz, H-7), 2.97 (1H, dd, *J* = 6.6 Hz, 16.3 Hz, H-7), 3.92 (3H, s, 1-OMe), 4.49 (1H, d, *J* = 14.1 Hz, H-9), 4.65 (1H, d, *J* = 14.1 Hz, H-9), 5.10 (1H, t, *J* = 7.1 Hz, H-6), 6.35 (1H, brs, H-10), 6.40 (1H, brs, H-12), 7.39 (2H, d, *J* = 8.8 Hz, H-14, H-18), 8.17 (2H, d, *J* = 8.8 Hz, H-15, H-17), ^13^C-NMR (150 MHz, CDCl_3_) δ: 6.9 (2-Me), 9.5 (4-Me), 24.1 (11-Me), 37.9 (C-7), 55.3 (1-OMe), 70.3 (C-9), 73.8 (C-6), 100.2 (C-2), 119.8 (C-10), 120.3 (C-4), 123.6 (C-15, C-17), 127.4 (C-12), 129.6 (C-14, C-18), 137.8 (C-11), 142.0 (C-8), 144.5 (C-13), 146.2 (C-16), 154.5 (C-5), 162.1 (C-1), 180.7 (C-3) [13].

### 2.4. Biological Activities

To exploit the antimicrobial activity of those isolated compounds, zones of growth inhibition against indicator bacteria were measured. Compound **9** and **6** clearly inhibited the growth of *Enterococcus faecalis* and *Staphylococcus aureus*, respectively (Figure S43). On the other hand, cytotoxic activities were examined for isolated compounds against three human tumor cell lines, Hela, lung cancer (A-549), and PC-3. As shown in Supplementary Figure S44, compounds **4, 7**, and **9** showed antiproliferative activity against Hela breast cancer cells, with around a 30% inhibition rate. Compounds **7**, **8**, **9**, and **10** showed antiproliferative activity against A-549 cancer cells.

## 3. Discussion

Rhizosphere microbial communities are an abundant reservoir of biological diversity in different ecosystems. To exploit bioactive natural products from these communities, we studied the microbial isolates from the rhizosphere of Clematidis Radix et Rhizoma (*Clematis manshurica* Rupr.) as an original plant used in a TCM. In this study, we focused on a specific *Streptomyces* isolate, *S. netropsis* WLXQSS-4, due to its noteworthy bioactivities. Genome sequencing and bioinformatics analysis revealed 40 BGCs, suggesting the potential of *S. netropsis* WLXQSS-4 as a biocontrol agent and biofertilizer in agriculture. For example, many genes in the *S. netropsis* WLXQSS-4 genome are associated with ammonia assimilation, phosphate solubilization, and indole-3-acetic acid (IAA) biosynthesis. Notably, IAA is beneficial on plant growth and pathogen defense [34,35] and ≈80% of bacteria isolated from the endophytic and rhizosphere of rice produce indolic compounds [36]. Furthermore, around 12% of the annotated genes in *S. netropsis* WLXQSS-4 were categorized as genes involved in amino acid metabolism (Figures S2 and S45–S46). Indeed, a range of tryptophan-derived metabolites were observed, including alloaurethin, indole, and pimprinine. Given the importance of tryptophan metabolism in plants, future field investigation is warranted to examine whether the strong tryptophan metabolism of *S. netropsis* WLXQSS-4 may contribute to the physiological metabolism of Clematidis Radix et Rhizoma (*Clematis manshurica* Rupr.), especially for its production of indole alkaloids [7].

Pimprinine is an indole alkaloid characterized with its oxazolindole ring moiety. Pimprinine derivatives have been found in many strains [37], and they display a wide range of biological activities [23,38,39,40,41]. In this study, a group of pimprinine congeners were purified. One derivative, N-acetyl-β-oxotryptamine (**9**), exhibited antiproliferative activities against human tumor cell lines and antibiotic activities against a Gram-positive pathogen, *Enterococcus faecalis*. Since the discovery of pimprinine in *Pseudomonas* [11], the biosynthetic mechanism of these oxazole molecules has remained elusive. Indolepyruvate or an unknown degradation product was proposed as a precursor of the indole moiety. Due to the difficulty of genetic manipulation, pimprinine biosynthesis has not been dissected through in vivo approach. Further efforts to unveil the biosynthetic mechanism of pimprinine in this rhizosphere microbe will be published in due course.

Through precursor feeding, we observed the biosynthesis of new halogenated pimprinine derivatives in *S. netropsis* WLXQSS-4. Precursor-directed biosynthesis incorporates non-native building blocks to generate “non-natural’’ natural products, but it requires a certain degree of promiscuity in gatekeeping enzymes such as adenylation and condensation domains. The isolation of new halogenated pimprinine derivatives in this study indicated not only the successful uptake of tryptophan analogues but also the relatively high promiscuity of pimprinine-synthesizing enzymes. Therefore, precursor feeding is proved feasible to broaden the product scope of *S. netropsis* WLXQSS-4, particularly for oxazoles compounds.

Together, we reported the genomic and metabolomic characterization of a TCM rhizosphere microbe, *S. netropsis* WLXQSS-4. In addition to elucidating the potential of microbial agents in agriculture of Clematidis Radix et Rhizoma, the work also laid a foundation in future biosynthetic investigation into primprnine and flazin molecules, which may be developed as potent antibacterial and antitumor agents. Moreover, new insights on the biosynthetic pathway of alloaureothin will assist the bioengineering of associated derivatives for drug development.

## 4. Materials and Methods

### 4.1. Isolation of Strain

*S. netropsis* WLXQSS-4 was isolated from the rhizosphere soil of *Clematrs mandshusica* Rupr. collected from Zhuanwanhe Village, Yingermen Town, Qingyuan County, Fushun City, Liaoning Province, China (28°5′59″ N, 115°3′19″ E). The strain was deposited at the Department of Natural Products Chemistry, School of Pharmacy, China Medical University with the voucher strain number WLXQSS-4.

For the strain purification, the rhizosphere soil was scraped into a sterilized plate and heated at 60 °C for 4 h to remove non-sporulating bacteria. Then, 0.5 g of soil was weighed and suspended in 5.0 mL of sterile water. After a series of dilution (10^−1^, 10^−2^, 10^−3^, 10^−4^, 10^−5^, 10^−6^), a 50 μL diluent was spread on SCK agar plates (soluble starch 10.0 g, KNO_3_ 2.0 g, casein 0.3 g, NaCl 2.0 g, K_2_HPO_4_ 2.0 g, MgSO_4_ 0.05 g, CaCO_3_ 0.02 g, FeSO_4_⋅7H_2_O 0.01 g, agar 15.0 g, demineralized water 1 L, adjusted pH to 7.8 with 2 N NaOH) added with nystatin (50 mg/L) and nalidixic acid (25 mg/L). After one week of cultivation at 28 °C;, visible colonies of actinomycetes were picked and streaked on new SCK agar plates. This purification process was repeated one time.

### 4.2. Morphological Identification

The isolate, *S. netropsis* WLXQSS-4, was further identified using various cultural characteristics including the growth optimization parameters on different medium.

Genus-level identification of the isolate was carried out based on aerial and substrate mycelium, reverse side pigmentation, and spore chain morphology following the Bergey’s Manual of Determinative Bacteriology. The arrangement of the spores in the mycelium was observed by the cover slip method under light microscope and by scanning electron microscope [42].

### 4.3. Fermentation in a Medium

After incubating on an SCK agar plate at 28 °C for 7 days, the strains were scraped and inoculated into 250 mL Erlenmeyer flasks containing 50 mL of liquid A medium consisting of soluble starch 20.0 g/L, glucose 10.0 g/L, peptone 5.0 g/L, yeast extract 5.0 g/L, NaCl 4.0 g/L, K_2_HPO_4_ 0.5 g/L, MgSO_4_.7H_2_O 0.5 g/L, CaCO_3_ 2.0 g/L, and demineralized water 1 L. The flasks were incubated at 28 °C with shaking (210 rpm) for 3 days to produce seed cultures. The seed culture (1.8 mL) was inoculated into 500 mL Erlenmeyer flask containing 100 mL of liquid A medium. The flasks were incubated at 28 °C with shaking (210 rpm) for 10 days.

### 4.4. Isolation of Secondary Metabolites (SM) from This Strain Fermentation Broth in a Medium

The fermentation broth (10 L) was centrifuged at 5000 rpm to obtain the mycelium and the supernatant. The mycelium was extracted three times with 500 mL MeOH and recovered organic solvent to give 40.96 g of crude extract. The supernatant was added with 4% (*w*/*v*) XAD-16N resin and shook on a rotary shaker (100 rpm) for 5 h. The mixed supernatant was filtrated and then washed with water and MeOH successively. MeOH eluate was collected and evaporated to yield 20.23 g of crude extract. The results of TLC and HPLC analysis showed that the two extracts had different compounds composition; thus, they were separated respectively.

The crude extract of supernatant was subjected to a HP-20 resin column chromatography using a gradient elution with MeOH−H_2_O (0:100−100:0) to yield six fractions (Fr.1−Fr.6). Fr.1 was subjected to open ODS column eluted with aquedous MeOH (20−100%) to give two subfractions (SubFr.1-1, SubFr.1-2). SubFr.1-1, which was eluted by aquedous MeOH (5−95%), was purified by semipreparative HPLC to obtain **5** (30.0 mg). SubFr.1-2 was purified by an open ODS column with an elution of aquedous MeOH (20−100%); then, it was followed by semipreparative HPLC eluted by aquedous MeOH (5−95%) to yield **9** (3.5 mg) and **7** (2.2 mg). Fr.4 was subjected to a Sephadex LH-20 column (MeOH) to give two subfractions (SubFr.4-1, SubFr.4-2). SubFr.4-1 was resolved in sequence by silica gel column (CH_2_Cl_2_−MeOH, 0:100−100:0), Sephadex LH-20 (MeOH), and semipreparative HPLC with aquedous MeOH (5−95%) to yield **3** (3.4 mg), **10** (3.0 mg) and **11** (4.0 mg). Compound **12** (1.2 mg) was isolated from SubFr.4-2 on semipreparative HPLC eluted with aqueous MeOH (5−95%). Fr. 5 was separated to a Sephadex LH-20 column (MeOH) two times to give **1** (2.7 mg).

The crude extract of mycelium was subjected to a HP-20 resin column chromatography eluted with MeOH−H_2_O (0:100−100:0) to give seven fractions (Fr.1−Fr.6). Fr.1 was chromatographed on silica gel with aquedous MeOH (20−100%) as an eluent to give two subfractions (SubFr.1-1, SubFr.1-2). Then, SubFr.1-1 was further purified by semipreparative HPLC to yield **8** (3.3 mg) with the eluent of aquedous MeOH (5−95%). Fr.5 was separated on a Sephadex LH-20 column by MeOH elution to yield two subfractions (SubFr.5-1, SubFr.5-2). SubFr.5-2 was further applied to a Sephadex LH-20 column in MeOH−CH_2_Cl_2_ (1:1) and semipreparative HPLC in aquedous MeOH (5−95%) to form compounds **2** (1.0 mg) and **4** (2.0 mg). Fr.6 was applied to semipreparative HPLC in aqueous MeOH (5−95%) to yield **6** (3.0 mg).

### 4.5. Fermentation in NL-19 Medium

After incubating on an SCK agar plate at 28 °C for 7 days, fully grown strains were scraped and inoculated into 250 mL Erlenmeyer flasks containing 50 mL of liquid NL-19 medium consisting of soybean flour 20.0 g/L, D-mannitol 20.0 g/L, and demineralized water 1 L, with an adjusted pH to 7.2 with 1 M HCl. The flasks were incubated at 28 °C with shaking (210 rpm) for 3 days to produce seed cultures. The seed culture (1.8 mL) was inoculated into a 500 mL Erlenmeyer flask containing 100 mL of liquid NL-19 medium. The flasks were incubated at 28 °C with shaking (210 rpm) for 5 days.

### 4.6. Isolation of SM from This Strain Fermentation Broth in NL-19 Medium

The fermentation broth (10 L) was treated using the same procedures as the introduction in the section of “Isolation of secondary metabolites (SM) from this strain fermentation broth in A medium”. Then, 41.2 g and 19.3 g extracts obtained respectively from the supernatant and mycelium.

The crude extract of supernatant was subjected to a HP-20 resin column chromatography eluted with MeOH−H_2_O (0:100−100:0) to give seven fractions (Fr.1−Fr.7). Fr.5 and Fr.6 were separated by semipreparative HPLC by gradient elution (aqueous MeOH, 5−95%) to form **13** (15.2 mg).

### 4.7. HR-LCMS Analysis and Generation of the Molecular Networking

HRESI MS analyses were executed on an UPLC system (Ultimate 3000, ThermoScientific, Germany) equipped with to a Thermo QExactive HF mass spectrometer (Thermo Fisher Scientific, US). The instrument was equipped with a Kinetex C18 column (50 cm × 2.1 mm, 100 Å).

A linear gradient analysis from 5% to 100% phase B was performed over 30 min with mobile phase A (H_2_O with 0.1% formic acid) and mobile phase B (acetonitrile with 0.1% formic acid). For each sample, 1 μL was injected onto the column at a flow rate of 0.3 mL/min.

The mass spectrometer was programmed to acquire MS/MS in a data-dependent manner, acquiring five MS/MS scans following each precursor MS_1_ scan.

The MS/MS raw data were converted to .mzXML format by MSConvert. A molecular network was created using the GNPS website (http://gnps.ucsd.edu, accessed on 4 March 2021) [9]. The molecular networking job on GNPS can be accessed at https://gnps.ucsd.edu/ProteoSAFe/status.jsp?task=87874391abea4f3ea8140c76f87a9574, accessed on 4 March 2021 and https://gnps.ucsd.edu/ProteoSAFe/status.jsp?task=45338a2a30514668b29735638d30a099, accessed on 4 March 2021. The data were filtered by removing all MS/MS fragment ions within +/−17 Da of the precursor *m*/*z*. MS/MS spectra were window filtered by choosing only the top 6 fragment ions in the +/−50Da window throughout the spectrum. The parent ion mass tolerance was fixed as 0.02 Da, and the MS_2_ ion fragment tolerance was set to 0.02 Da. The cosine score was adopted to evaluate the similarity of the MS_2_ spectra; then, the network was generated based on the cosine score (1 indicates identical spectra, while 0 indicates no similarity). In our case, the network threshold was set as the cosine score above 0.3 and at least 1 matched peak.

### 4.8. Scanning Electron Microscopy

The sample firstly was prepared as protocol described previously [43]. The spores of *S. netropsis* WLXQSS-4 were collected, washed with phosphate-buffered saline (PBS), and fixed with 3% glutaraldehyde at 4 °C overnight. The fixed mycelium was washed with PBS three times (15 min each) and then fixed with 1% OsO4 for 1 h. Subsequently, an ethanol concentration gradient (v/v) of 30%, 50%, 90%, and 100% was used to dehydrate the fixed mycelia sequentially. Morphological characteristics of the mycelia surface were examined using a JSM-5410LV scanning electron microscopy (JEOL, Japan) [44].

### 4.9. Genome Sequencing and Annotation

The gene encoding 16S rRNA was amplified using two universal primers (pA and pH) [45]. The sequencing result revealed a high sequence similarity (100%, 1465/1466) between *S. netropsis* NBRC 12,893 and *S. netropsis* NBRC 3723 as the closest homologous strain. *S. netropsis* WLXQSS-4 was cultivated in 20 mL TSB medium (Tryptic Soy Broth 30 g, demineralized water 1 L, with an adjusted pH to 7.2 with 2 N NaOH) at 28 °C for 2 days on a rotary shaker at 180 r·min^−1^. Cells were pelleted by centrifugation, and genomic DNA were extracted using the methods described by Zhang et al. [46]. The whole genome was sequenced using a combination of Illumina Hiseq and Pacific Bioscience SMRT (PacBio RSII) sequencing platform, with 601-fold average genome coverage. The genome sequencing, assembly, and basic bioinformatics analysis of *S. netropsis* WLXQSS-4 were performed by MAGIGENE Co., Ltd. A total of 28,387,120 Rawreads from Illumina sequencing data were assembled de novo by the SOAPdenovo (v2.04) method [45]. The PacBio sequencing data were corrected by mapping the Illumina sequencing reads on BLASR (Basic Local Alignment with Successive Refinement) and then assembled by the Celera Assembler (http://wgs-assembler.sourceforge.net, accessed on 4 March 2021). After generating a reliable scaffold, correction of sequencing reads was performed again based on the Illumina data. Sequencing quality control on raw sequence data were checked by FastQC (http://www.bioinformatics.babraham.ac.uk/projects/fastqc/, accessed on 4 March 2021).

Putative protein-coding sequences were predicted based on results from GLIMMER 3.02 (https://ccb.jhu.edu/software/glimmer/, accessed on 4 March 2021). The coding sequences were further annotated using the stand-alone version of HMMER v3.1b2 (http://hmmer.org/, accessed on 4 March 2021) and by downloading all HMM models for bacteria from eggNOG v4.5.0. Additional analysis was carried out using the UniProt database (http://www.uniprot.org/, accessed on 4 March 2021), the RAST database (https://rast.nmpdr.org/, accessed on 4 March 2021), and Cluster of Orthologous Group of Proteins (https://www.ncbi.nlm.nih.gov/COG/, accessed on 4 March 2021) [47]. rRNA and tRNA genes were predicted with RNAmmer-1.2 [48] and tRNA scan-SE [49]. AntiSMASH (antiSMASH 5.0) (https://antismash.secondarymetabolites.org, accessed on 4 March 2021) and Prism (https://prism.adapsyn.com/, accessed on 4 March 2021) were used to predict the gene clusters that may have potential for the production of secondary metabolites, which was followed by careful manual correction [50,51].

### 4.10. Cytotoxicity Assay

Three tested human tumor cell lines, human leukemia (HL60), lung cancer (A549), and human prostate cancer (PC-3) were purchased from ATTC (Manassas, VA, USA). Camptothecin was used as a positive control.

Each of these cell lines was incubated in medium DMEM or RPMI-1640 containing 10% fetal bovine serum at 37 °C under humidified atmosphere with 5% CO_2_. The cytotoxicity of the isolates toward these tumor cell lines was assessed via the 3-(4, 5-dimethylthiazol-2-yl)-5(3-carboxymethoxyphenyl)-2-(4sulfopheny)-2H tetrazolium (MTS) (Promega, Madison, WI, USA) method. Camptothecin was used as a positive control.

The cell lines were inoculated into each well of the normal 96-well plates and incubated for 12 h before the addition of the test compounds. Different concentrations of each compound were added and exposed to the cells for a continuous cultivation of 48 h. The isolates with inhibition rates ≥50% against the cell lines were further assessed in triplicate at different concentrations (0.064, 0.32, 1.6, 8, and 40 µM). The IC_50_ values were measured based on Reed and Muench’s method [52]. All the experiments were carried out in triplicate.

### 4.11. Antibacterial Activity Assay

Antibacterial in vitro assay was performed to test the antagonistic effects of the isolated strain *S. netropsis* WLXQSS-4 against pathogenic bacteria, including *Staphylococcus aureus*, *Bacillus cereus*, and *Enterococcus faecalis* (laboratory collection). The antibacterial bioassays were carried out based on the Kirby–Bauer Disk Diffusion Susceptibility Test as described by Kirby–Bauer disk diffusion susceptibility test protocol. Ampicillin and kanamycin were used as positive control, while methanol solvent was used as negative control. The initial sample concentration was set as 1 mg/mL. The experiment was repeated three times.

## Figures and Tables

**Table 1:** General features of the chromosome of *S. netropsis* WLXQSS-4.

Features	Chromosome Characteristics
Genome topology	Linear
Chromosome size (bp)	8,324,019
Scaffolds	3
G+C content (%)	71.32
Protein-coding genes	7115
Gene density (per kb)	0.85
Average ORF size (bp)	1018.05
rRNA (16S-23S-5S) operons	21
sRNA	1
Number of tRNA	78
Secondary metabolites gene cluster	40

## Data Availability

The data presented are available in the manuscript and supplementary materials.

## Supplementary Materials

The following are available online, Table S1: Secondary metabolites gene clusters identified in *S. netropsis* WLXQSS-4, Table S2: Deduced functions of ORFs in the alloaureothin BGC from *S. netropsis* WLXQSS-4, Figure S1: The complete genome of rhizosphere Streptomyces Isolates *S. netropsis* WLXQSS-4, Figure S2: Assignment of 4047 genes of *S. netropsis* WLXQSS-4 to the functional groups of the actNOG subset of the eggNOG database, Figure S3: MS spectrum of **1**, Figure S4: ^1^H-NMR spectrum of **1**, Figure S5: ^13^C-NMR spectrum of **1**, Figure S6: ^1^H-NMR spectrum of **2**, Figure S7: ^13^C-NMR spectrum of **2**, Figure S8: MS spectrum of **3**, Figure S9: ^1^H-NMR spectrum of **3**, Figure S10: ^13^C-NMR spectrum of **3**, Figure S11: ^1^H-NMR spectrum of **4**, Figure S12: ^13^C-NMR spectrum of **4**, Figure S13: ^1^H-NMR spectrum of **5**, Figure S14: ^13^C-NMR spectrum of **5**, Figure S15: ^1^H-NMR spectrum of **6**, Figure S16: ^13^C-NMR spectrum of **6**, Figure S17: HR-MS spectrum of **7**, Figure S18: ^1^H-NMR spectrum of **7**, Figure S19: ^13^C-NMR spectrum of **7**, Figure S20: ^1^H-NMR spectrum of **8**, Figure S21: ^13^C-NMR spectrum of **8**, Figure S22: MS spectrum of **9**, Figure S23: ^1^H-NMR spectrum of **9**, Figure S24: ^13^C-NMR spectrum of **9**, Figure S25: MS spectrum of **10**, Figure S26: ^1^H-NMR spectrum of **10**, Figure S27: ^13^C-NMR spectrum of **10**, Figure S28: ^1^H-NMR spectrum of **11**, Figure S29: ^13^C-NMR spectrum of **11**, Figure S30: HR-MS spectrum of **12**, Figure S31: ^1^H-NMR spectrum of **12**, Figure S32: ^13^C-NMR spectrum of **12**, Figure S33: HR-MS spectrum of compound 6′-chloropimprinine, Figure S34: HR-MS spectrum of compound 6′-chloropimprinol A, Figure S35: HR-MS spectrum of compound 6′-chloropimprinethine, Figure S36: HR-MS spectrum of compound 6′-bromopimprinethine, Figure S37: The possible biosynthesis pathway of pimprinine derivatives, Figure S38: Two postulated pathways for the biosynthesis of β-carboline compound flazin, **Figure S39**: MS spectrum of **13**, Figure S40: HR-MS spectrum of compound **13**, Figure S41: ^1^H-NMR spectrum of **13**, Figure S42: ^13^C-NMR spectrum of **13**, Figure S43: The antibacterial assay against *Staphylococcus aureus*, *Bacillus cereus*, and *Enterococcus faecalis*, Figure S44: MTT assay to determine compound (**1**–**12**) cytotoxicity against hela, lung cancer (A-549), and PC-3, Figure S45: KEGG pathway annotation statistics of *S. netropsis* WLXQSS-4, Figure S46: KEGG pathway related with the tryptophan metabolism in *S. netropsis* WLXQSS-4.

## Sample Availability

Samples of the compounds are not available from the authors.
