# Supplementary material for: Genomic and Metabolomic Investigation of a Rhizosphere Isolate Streptomyces netropsis WLXQSS-4 Associated with a Traditional Chinese Medicine
Source: Molecules. 2021 Apr 8;26(8):2147. doi: 10.3390/molecules26082147 (PMC8068340; doi:10.3390/molecules26082147)
Supplement: Supplementary file 1 [file molecules-26-02147-s001.pdf]

molecules

Supplementary Material

Genomic and metabolomic investigation of a rhizosphere isolate *Streptomyces netropsis* WLXQSS-4 associated with a traditional Chinese medicine

Songya Zhang [1], Lingxiao Zhang [2], Jing Zhu [1], Hong Chen [2], Zhicong Chen [1], Tong Si [1,*] and Tao Liu [2,*]

[1] Shenzhen Institute of Synthetic Biology, Shenzhen Institutes of Advanced Technology, Chinese Academy of Sciences, 518055 Shenzhen, China; sy.zhang2@siat.ac.cn (S.Z.); jing.zhu@siat.ac.cn (J.Z.); zc.chen@siat.ac.cn (Z.C.)
[2] Department of Natural Products Chemistry, School of Pharmacy, China Medical University, 110122 Shenyang, China; 13022551727@163.com (L.Z.); chenhong950914@163.com (H.C.)
* Correspondence: liutao@cmu.edu.cn (T.L.); tong.si@siat.ac.cn (T.S.)

Supplementary Tables and Figures

Table 1. Secondary metabolites gene clusters identified in *S. netropsis* WLXQSS-4.

No	Region	BGC Type	From	To	Most similar known cluster	Similarity
1	Region 1.1	indole	51,478	71,825		
2	Region 1.2	terpene	603,609	623,506		
3	Region 1.3	lanthipeptide	644,254	666,487	glycinocin A	9%
4	Region 1.4	terpene	672,388	692,712		
5	Region 1.5	siderophore	700,276	710,224	desferrioxamine E	100%
6	Region 1.6	other	745,160	786,533	A-503083 A	7%
7	Region 1.7	siderophore	1,581,615	1,593,564		
8	Region 1.8	terpene	1,924,713	1,945,053		
9	Region 1.9	T3PKS	2,120,118	2,160,713	chloropentostatin	9%
10	Region 1.10	linaridin	2,474,856	2,495,008	legonaridin	50%
11	Region 1.11	ladderane	2,643,237	2,700,700	vazabitide A	28%
12	Region 1.12	butyrolactone	3,091,492	3,102,556	griseoviridin	8%
13	Region 1.13	T1PKS	3,449,880	3,498,595	berninamycin A	33%
14	Region 1.14	melanin	4,227,817	4,236,356	melanin	28%
15	Region 1.15	melanin	4,450,489	4,460,905	melanin	28%
16	Region 1.16	linaridin	4,533,015	4,553,593	pentostatine / vidarabine	12%
17	Region 1.17	lanthipeptide	4,572,452	4,595,100	SapB	100%
18	Region 1.18	ectoine	5,025,826	5,036,224	ectoine	100%
19	Region 1.19	transAT-PKS	5,437,035	5,551,637	calyculin A	32%
20	Region 1.20	T2PKS	5,671,575	5,756,808	JBIR-76 / JBIR-77	68%
21	Region 1.21	terpene	5,884,803	5,904,975	guadinomine	7%
22	Region 1.22	T1PKS	6,059,563	6,133,399	borrelidin	52%
23	Region 1.23	terpene	6,153,042	6,174,256		
24	Region 1.24	NRPS	6,176,361	6,228,931	streptobactin	47%
25	Region 1.25	T3PKS	6,332,926	6,368,849	violapyrone B	28%
26	Region 2.1	terpene	64,971	84,230		
27	Region 2.2	lassopeptide	139,695	162,389	lagmysin	80%
28	Region 2.3	NRPS	213,201	293,484	vazabitide A	17%
29	Region 2.4	NRPS	301,963	351,870	enduracidin	4%

**30**	Region 2.5	NRPS	398,765	467,523	salinosporamide A	45%
**31**	Region 2.6	T1PKS	534,821	664,616	aureothin	100%
**32**	Region 2.7	T1PKS	665,727	833,898	nystatin A1	72%
**33**	Region 2.8	NRPS	866,511	918,299	deimino-antipain	66%
**34**	Region 2.9	NRPS	981,848	1,101,191	netropsin	100%
**35**	Region 2.10	siderophore	1,441,513	1,455,189		
**36**	Region 2.11	terpene	1,488,508	1,515,160	hopene	76%
**37**	Region 2.12	indole	1,542,279	1,565,584	AT2433-A1	14%
**38**	Region 2.13	butyrolacton e	1,603,139	1,613,909		
**39**	Region 2.14	NRPS	1,638,931	1,716,293	bacillibactin	46%
**40**	Region 2.15	NRPS	1,853,481	1,947,344	atratumycin	55%

Table 2. Deduced functions of ORFs in the alloaureothin BGC from *S. netropsis* WLXQSS-4.

Gene	Size (a.a)	Protein homologue (accession number)	Identity (%)	Proposed Function
**Orf(+2)**	500	WP_184731891.1	98.9	carboxylesterase family protein
**Orf(+1)**	297	WP_184731894.1	97.6	polysaccharide deacetylase family protein
**aluD**	273	aurD (CAE02599.1)	90	AurD protein /transcriptional regulator
**aluE**	505	aurE (CAE02600.1)	91	p-nitrobenzoate CoA ligase
**aluF**	338	aurF(CAE02601.1)	83	p-aminobenzoate N-oxygenase
**aluA**	1920	aurA (CAE02602.1)	79	polyketide synthase type I
**aluG**	701	aurG (CAE02603.1)	80	PABA synthase
**aluH**	410	aurH (CAE02604.1)	81	bifunctional cytochrome P450 monooxygenase
**aluB**	2183	aurB (CAE02605.1)	77	polyketide synthase type I
**aluC**	2282	aurC (CAE02606.1)	75	polyketide synthase type I
**aluI**	229	aurI (CAE02607.1)	77	O-methyl transferase
**Orf(-1)**	432	WP_184731913.1	97.4	siderophore 2,3-dihydroxybenzoate-glycine-threonine trimeric ester bacillibactin synthetase
**Orf(-2)**	339	WP_184732233.1	98.7	LuxR family transcriptional regulator
**Orf(-3)**	449	WP_184732235.1	99.5	MFS transporter

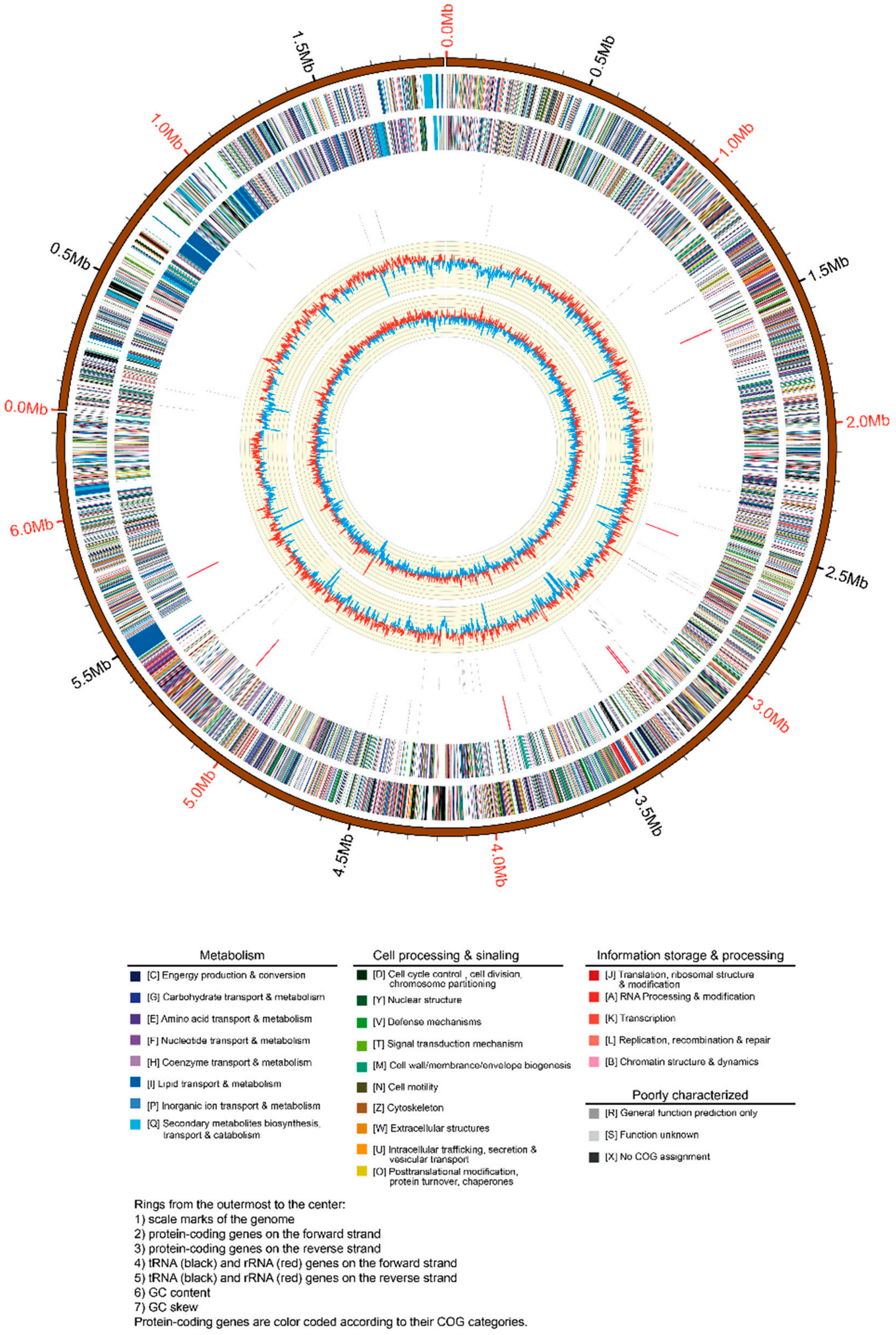

Figure 1. The complete genome of rhizosphere Streptomyces Isolates *S. netropsis* WLXQSS-4. The five circles (outer to inner) represent forward strand CDSs, reverse strand CDSs, nomenclature, and locations of predictive secondary metabolites generated using antiSMASH 3.0 software, GC content and GC skew. Putative alloaurethin cluster herein referred to cluster *alu*.

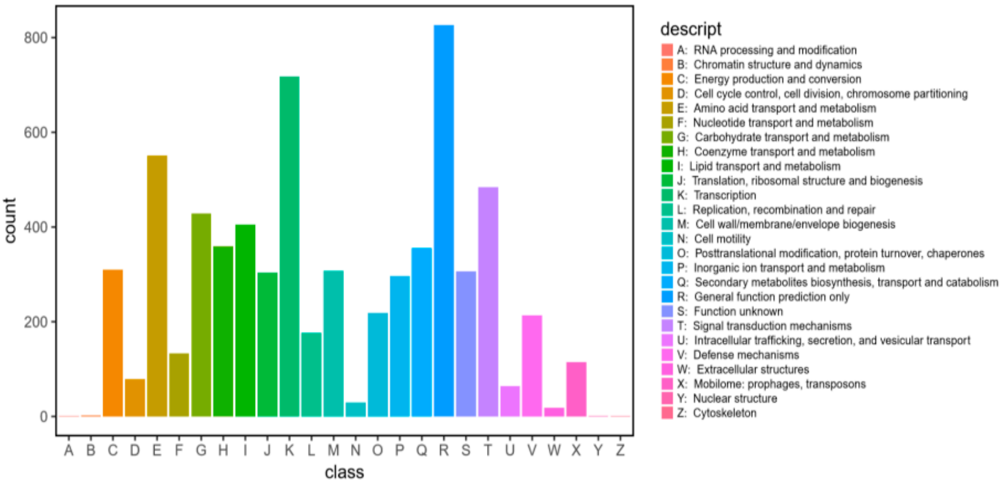

Figure 2. Assignment of 4047 genes of *S. netropsis* WLXQSS-4 to the functional groups of the actNOG subset of the eggNOG database.

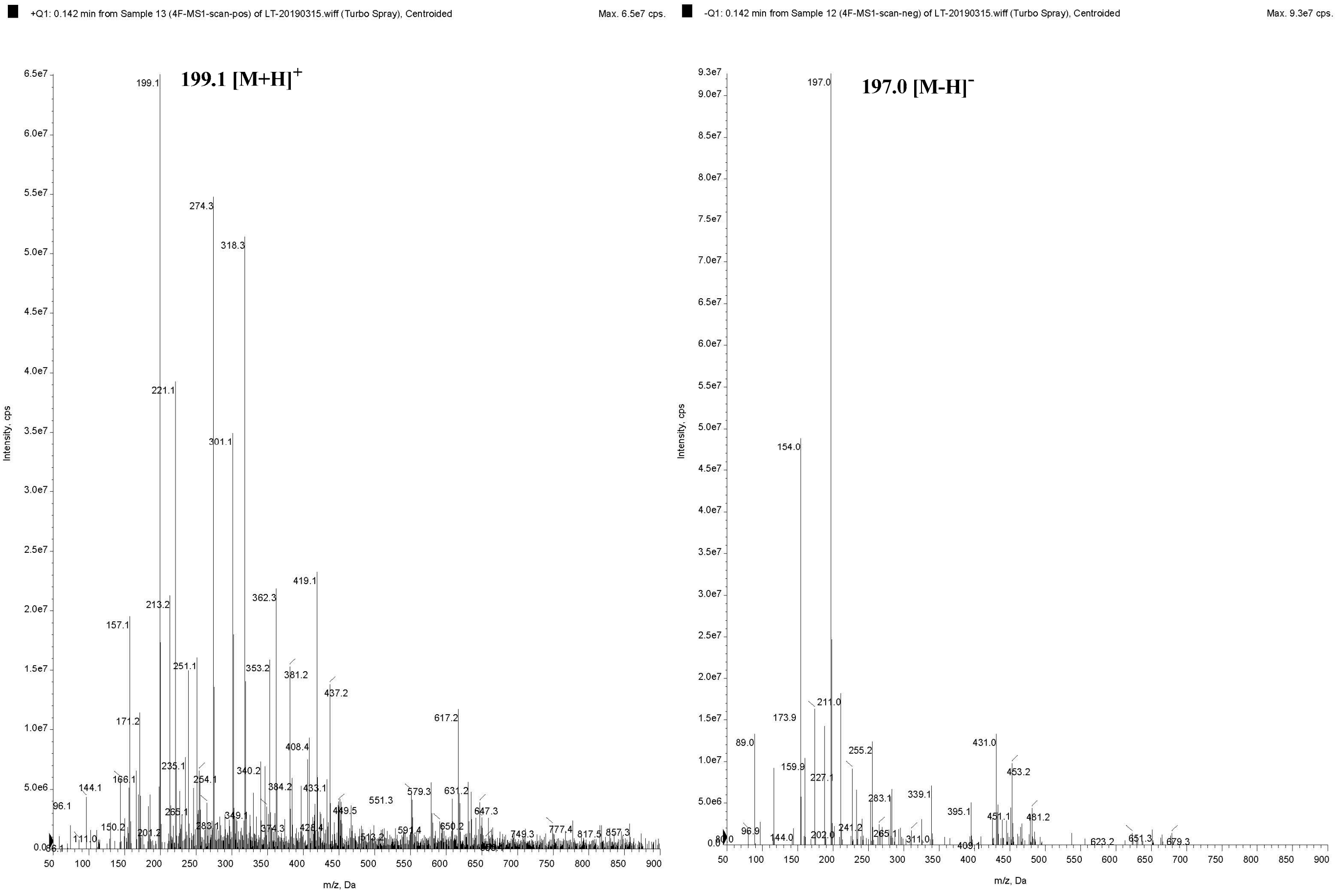

Figure 3. MS spectrum of **1.**

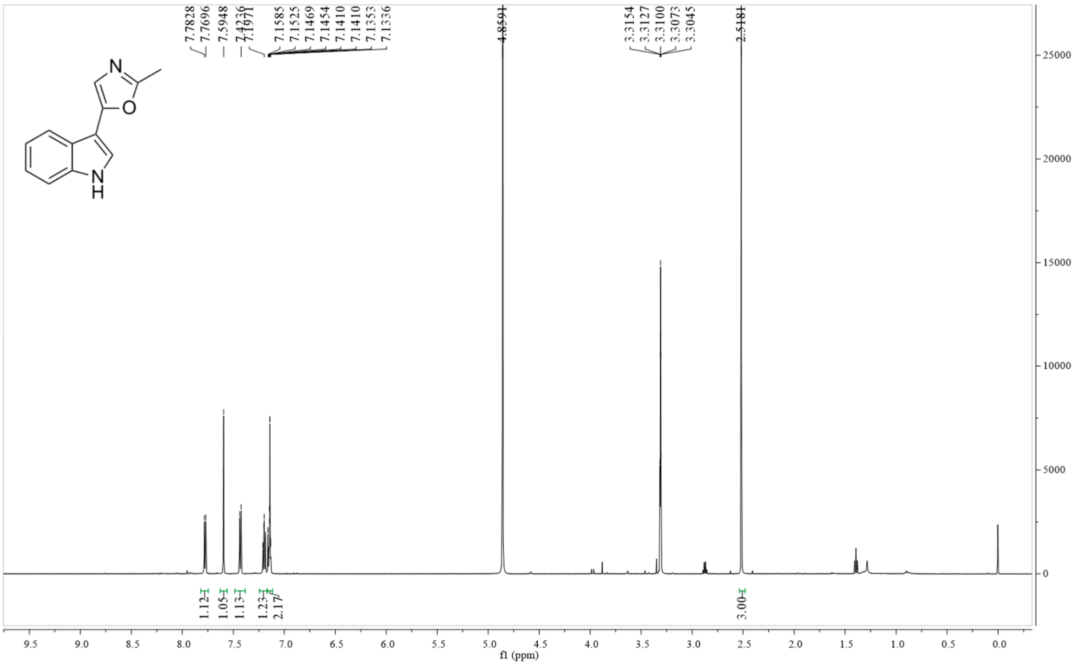

Figure 4. ^{1}H-NMR spectrum of **1.**

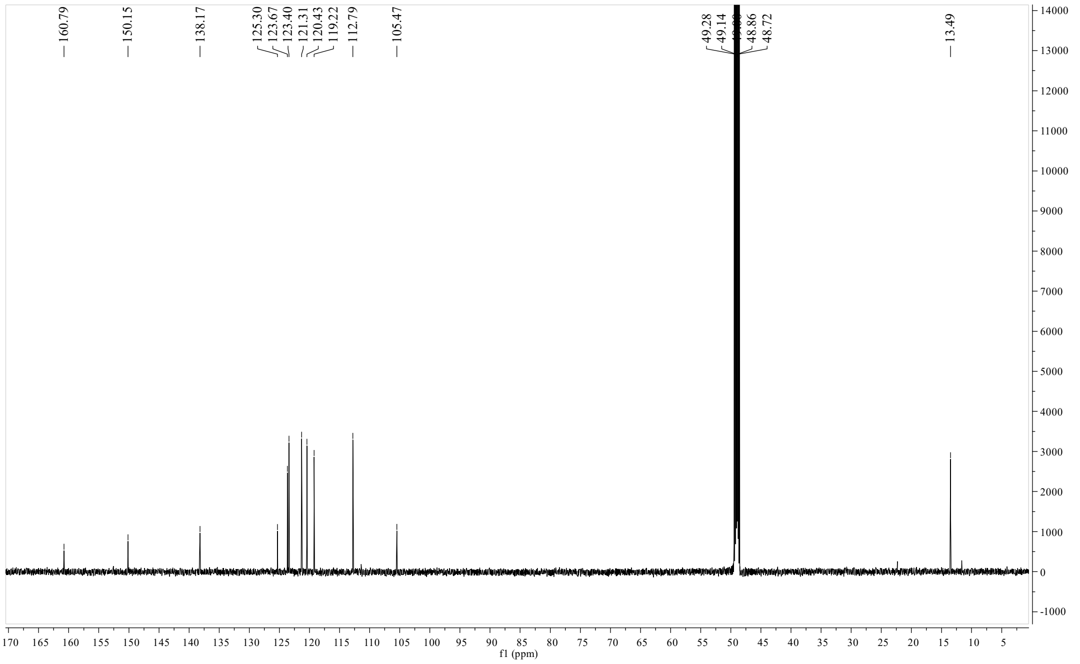

Figure 5. ^{13}C-NMR spectrum of **1.**

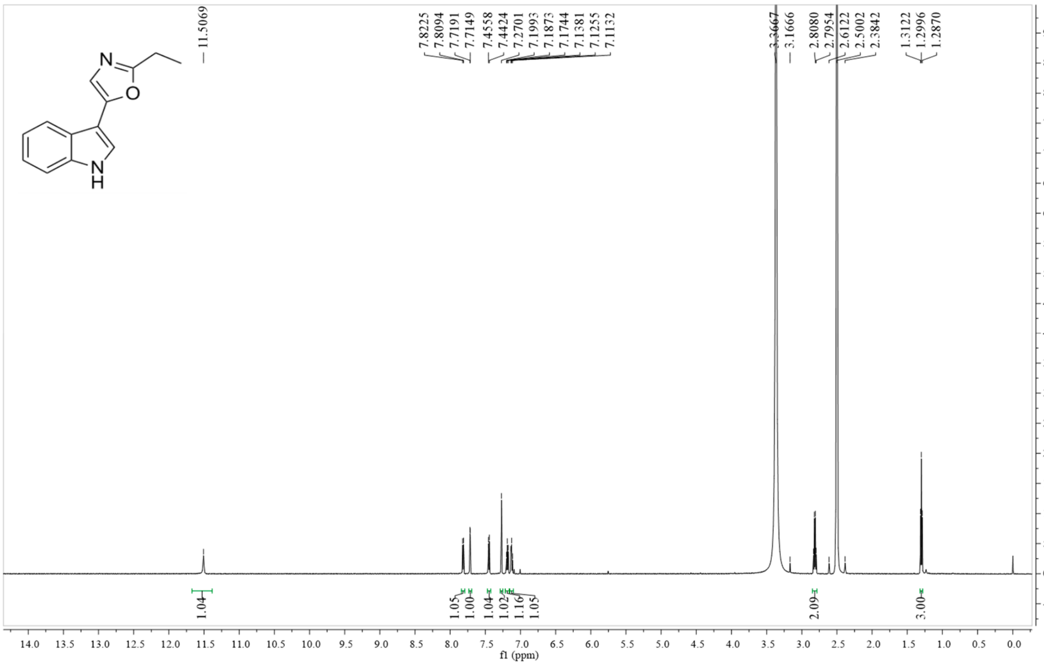

Figure 6. ^{1}H-NMR spectrum of **2.**

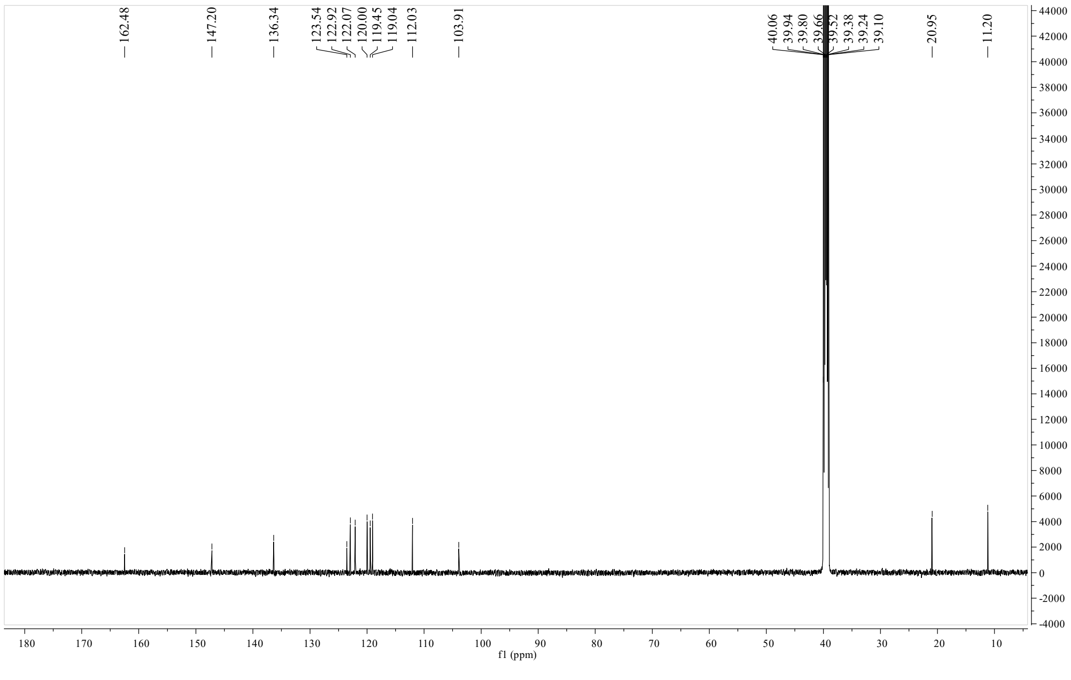

Figure 7. ^{13}C-NMR spectrum of **2.**

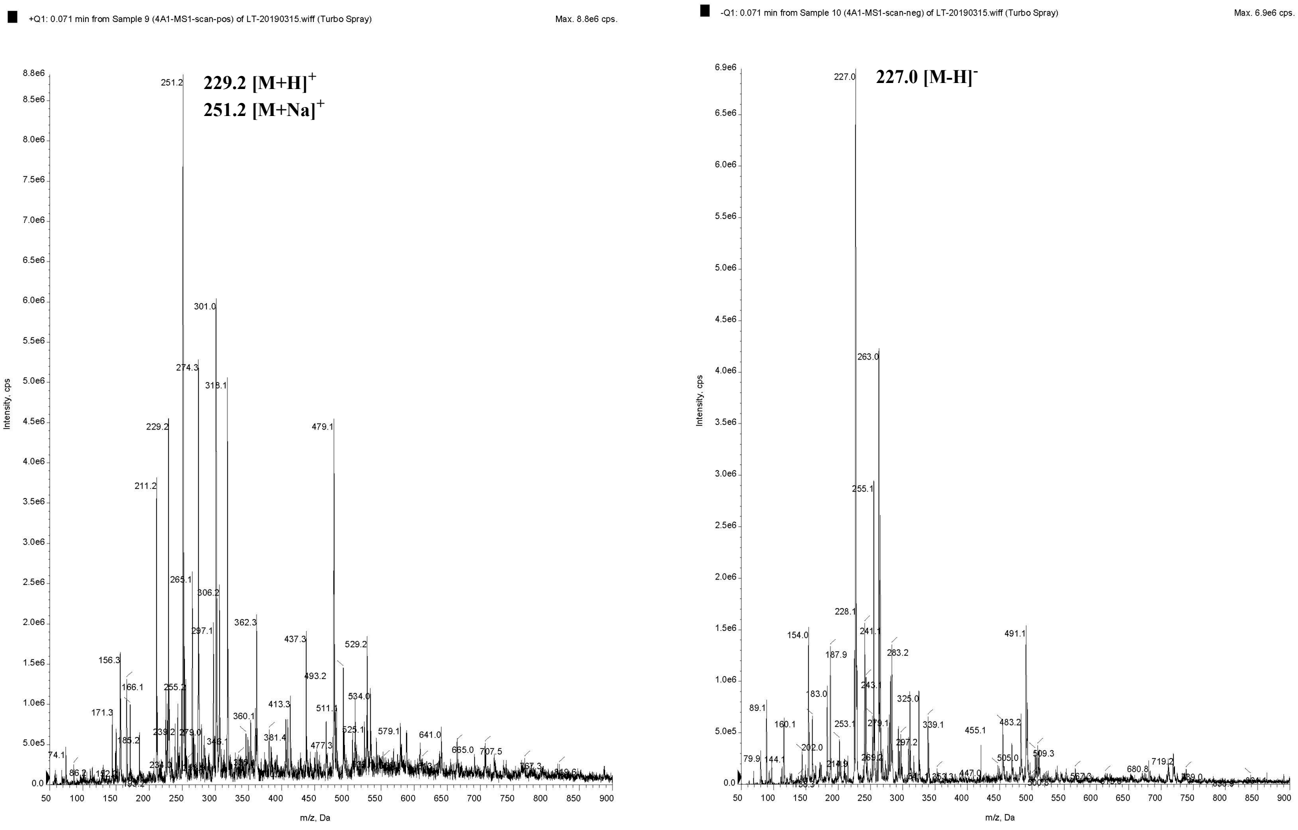

Figure 8. MS spectrum of **3.**

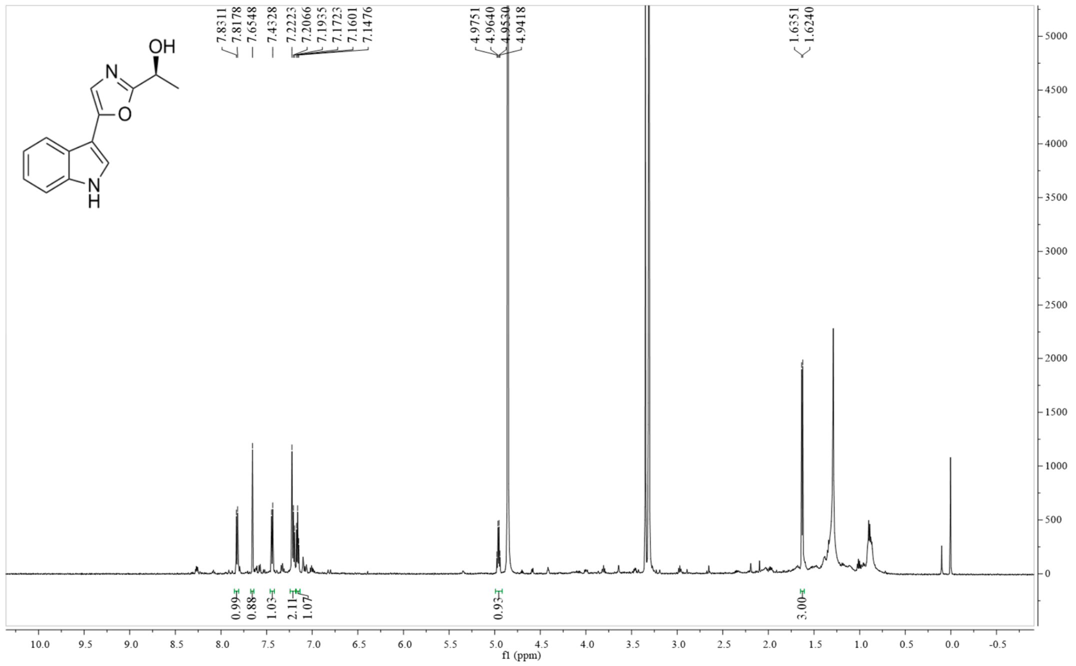

Figure 9. ^{1}H-NMR spectrum of **3.**

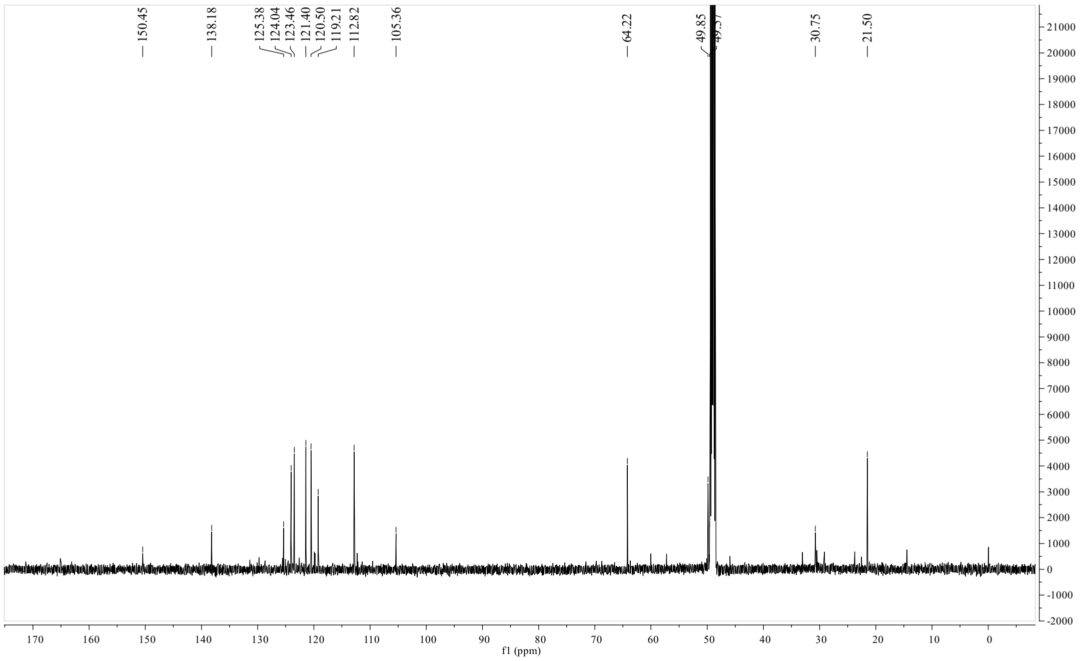

Figure 10. ^{13}C-NMR spectrum of **3.**

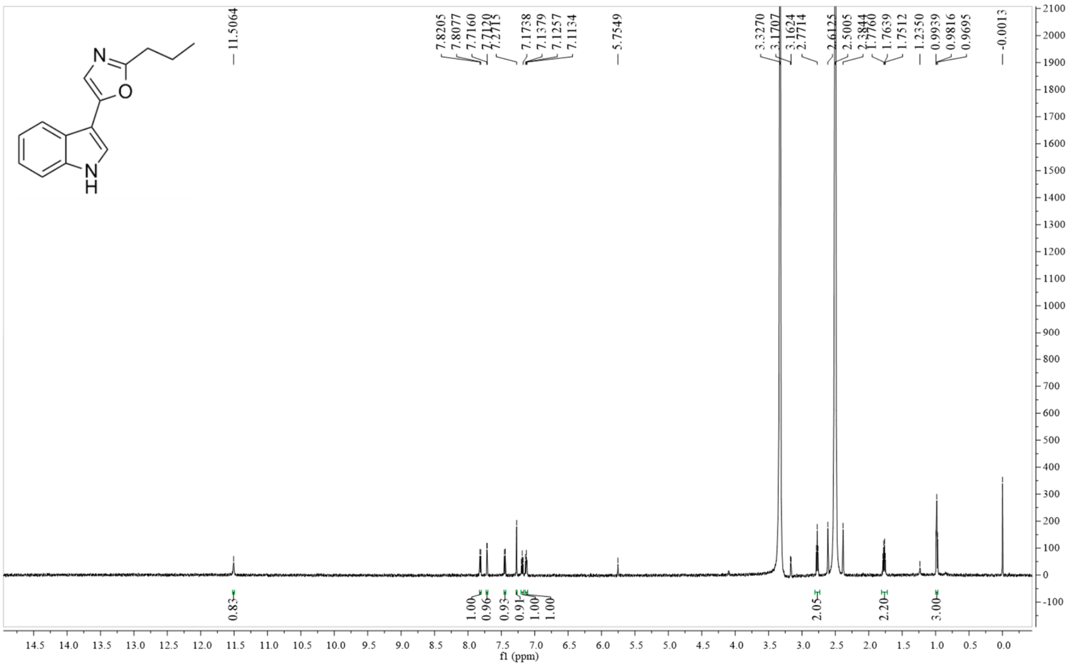

Figure 11. ^{1}H-NMR spectrum of **4.**

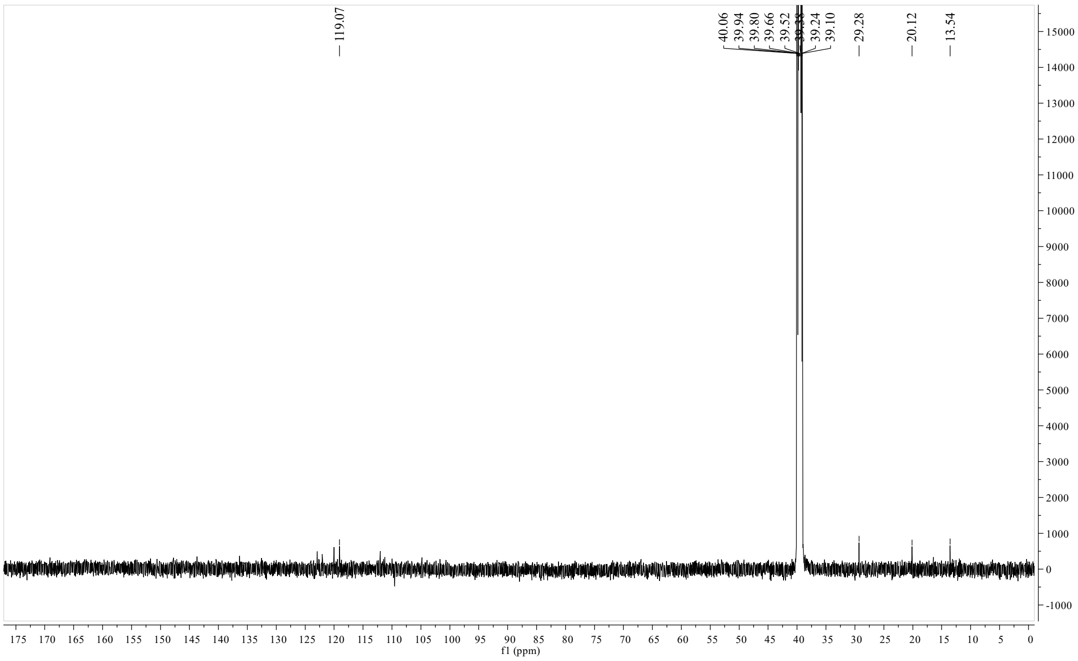

Figure 12. ^{13}C-NMR spectrum of **4.**

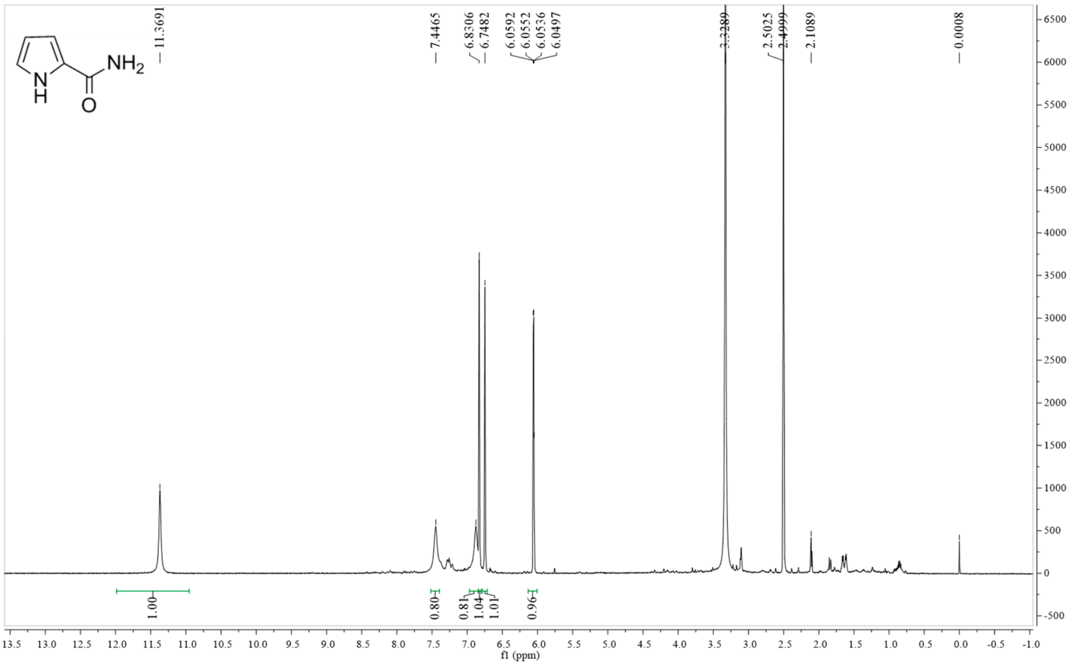

Figure 13. ^{1}H-NMR spectrum of **5.**

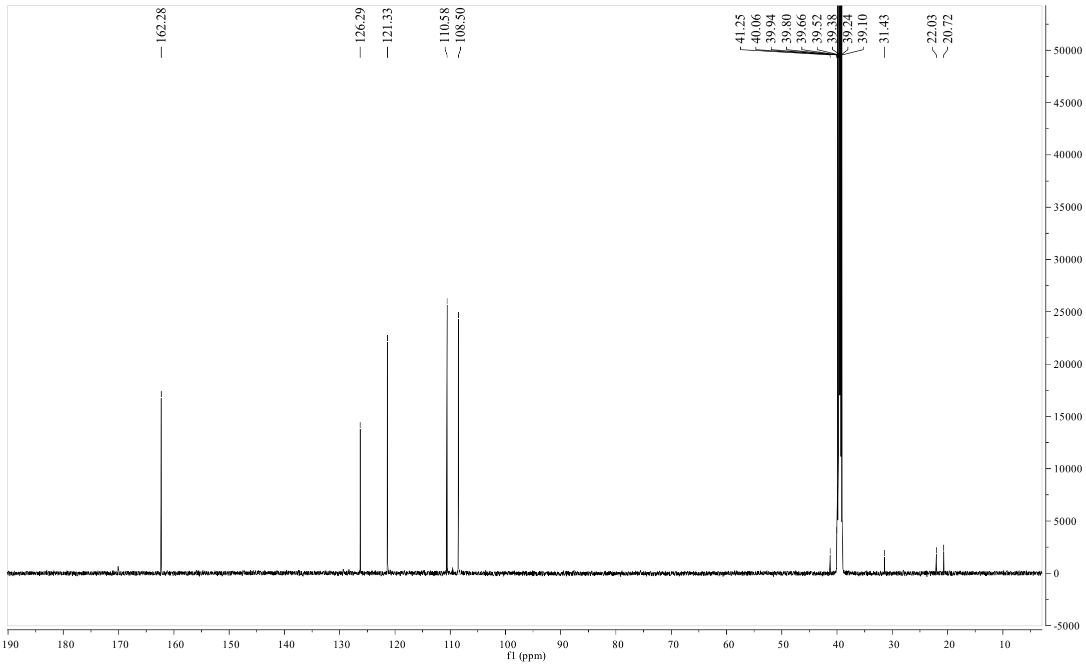

Figure 14. ^{13}C-NMR spectrum of **5.**

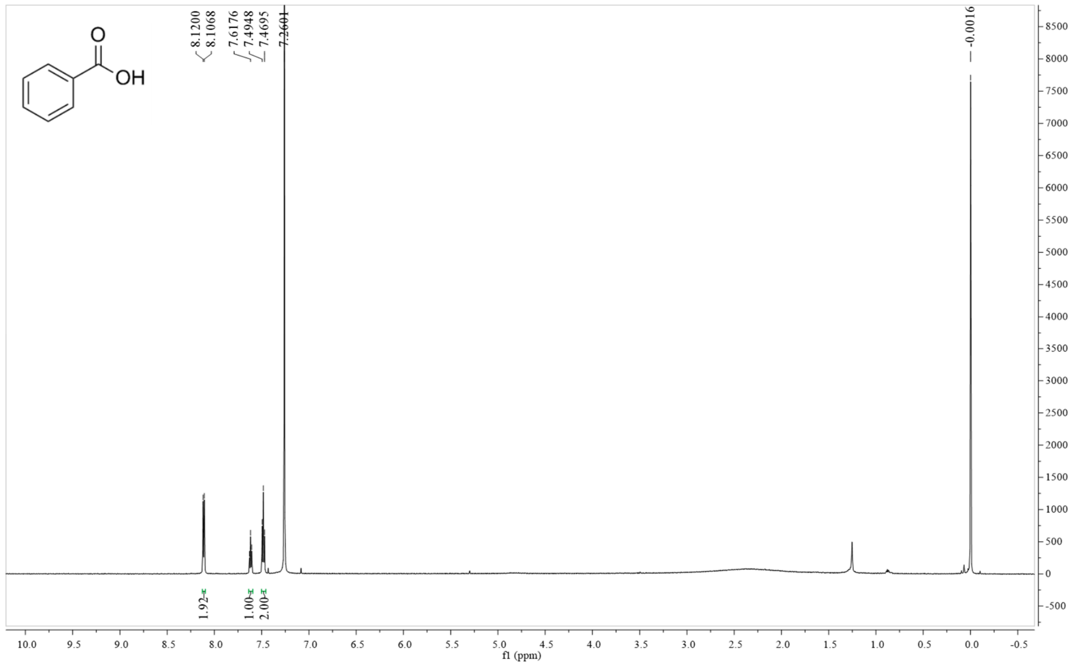

Figure 15. ^{1}H-NMR spectrum of **6.**

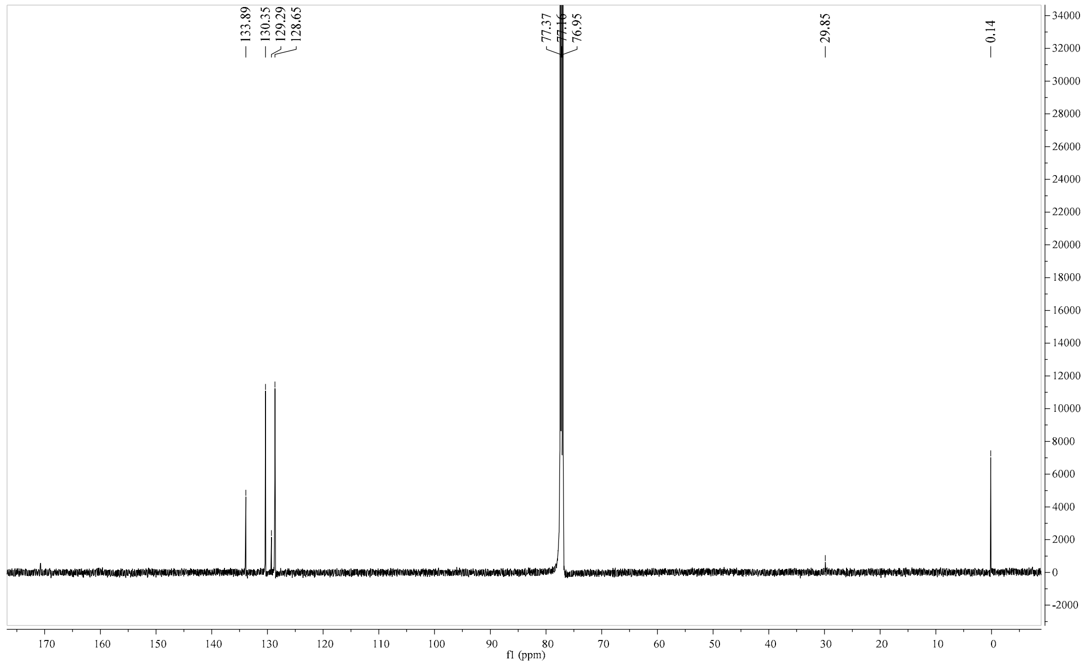

Figure 16. ^{13}C-NMR spectrum of **6.**

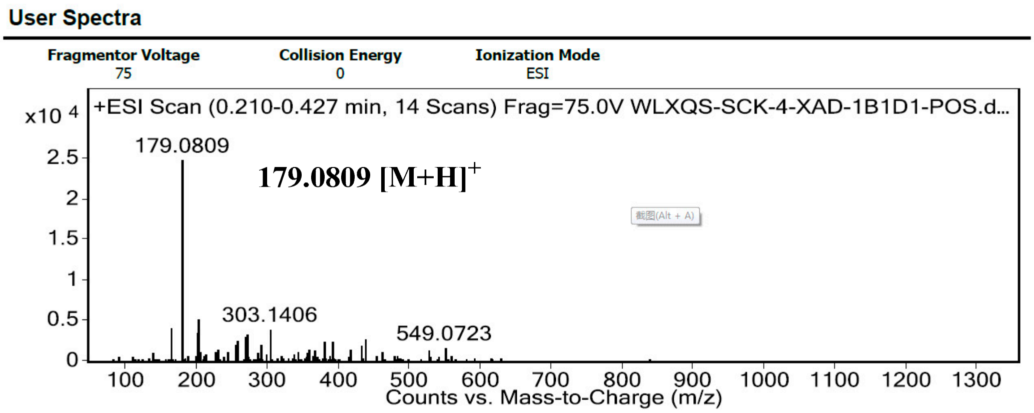

Figure 17. HR-MS spectrum of **7.**

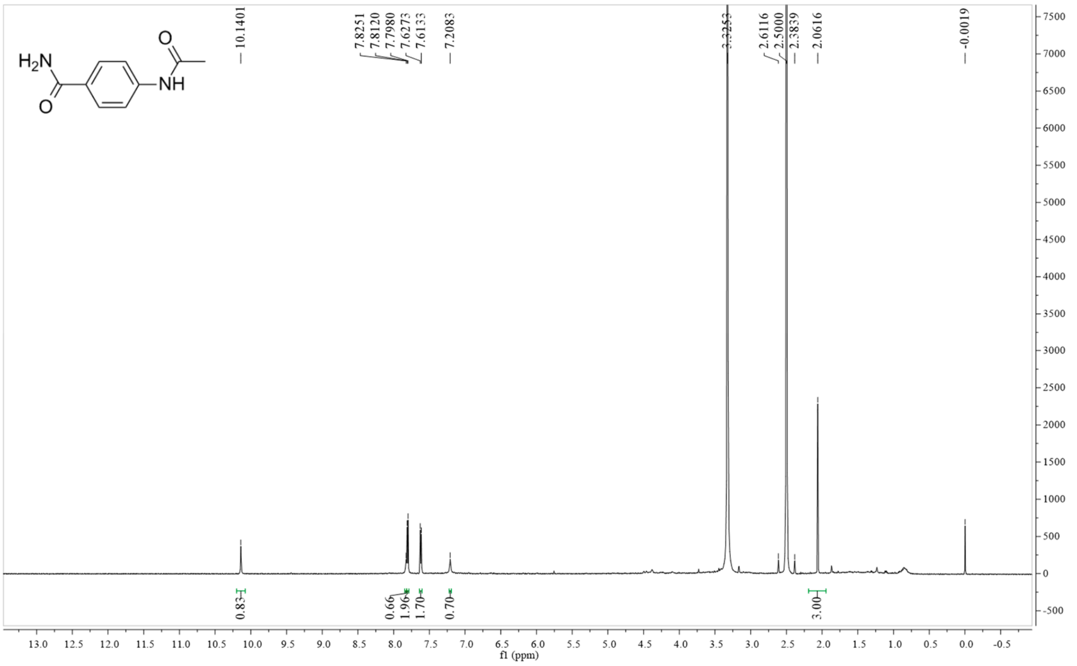

Figure 18. ^{1}H-NMR spectrum of **7.**

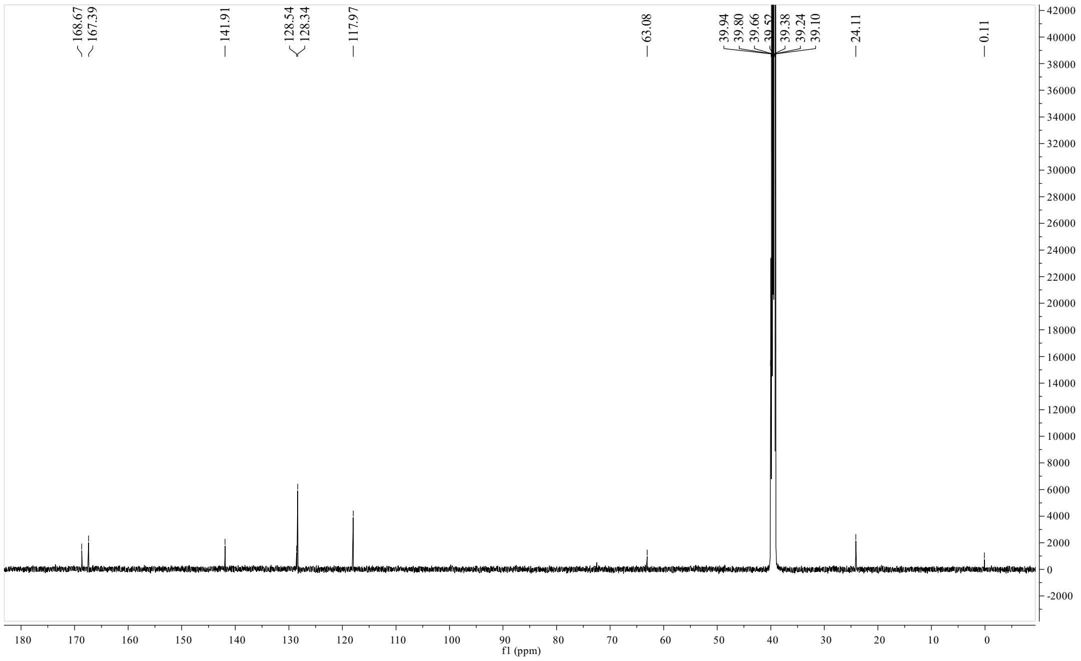

Figure 19. ^{13}C-NMR spectrum of **7.**

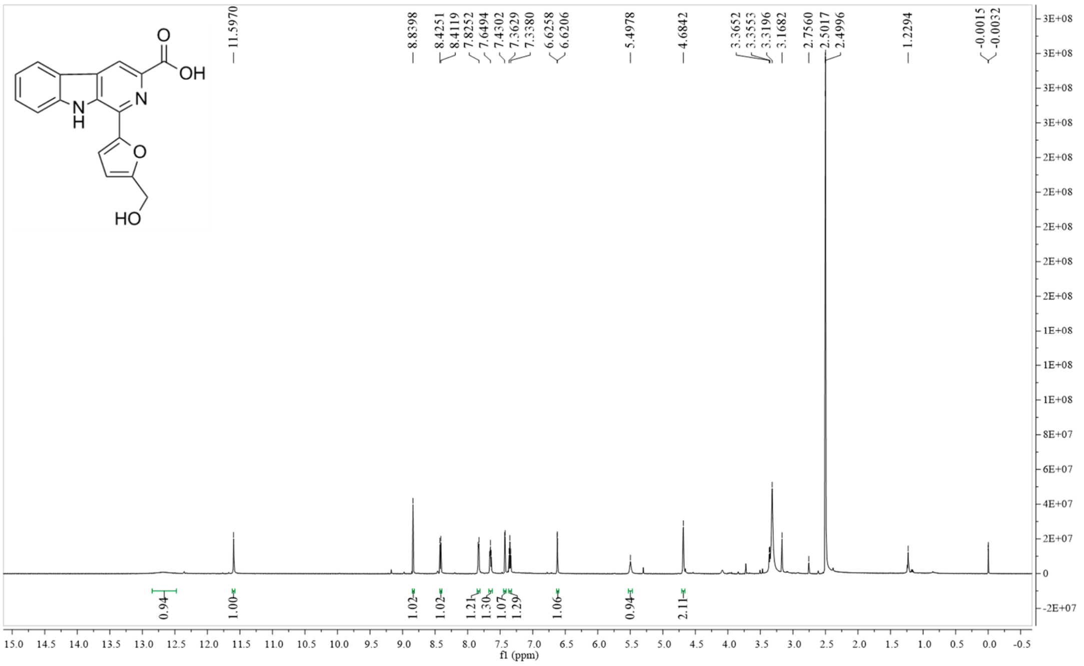

Figure 20. ^{1}H-NMR spectrum of **8.**

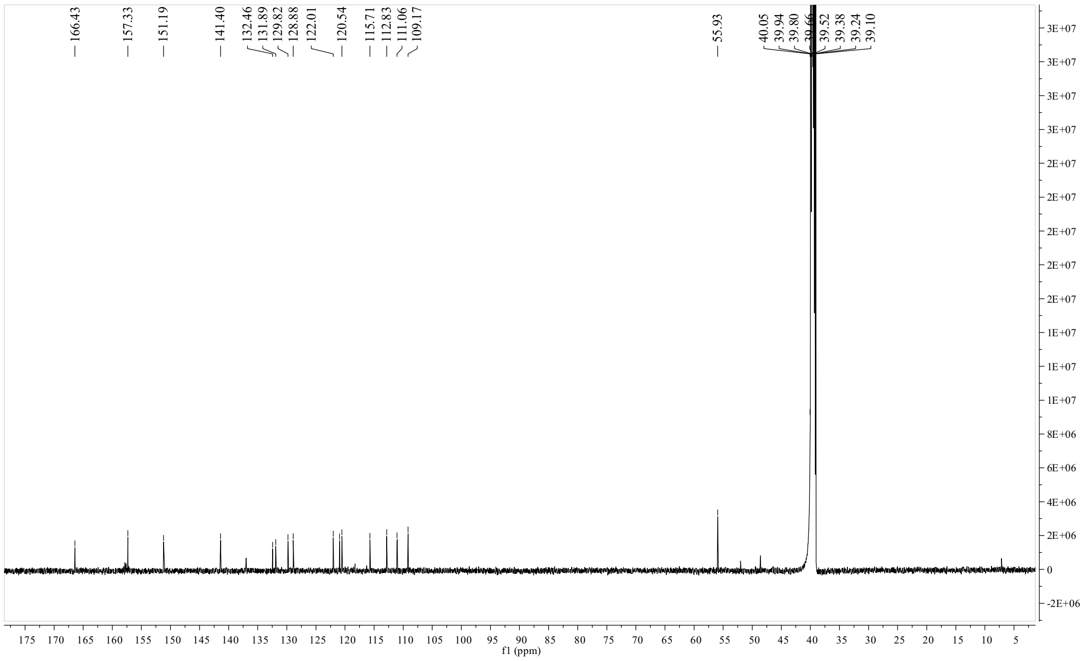

Figure 21. ^{13}C-NMR spectrum of **8.**

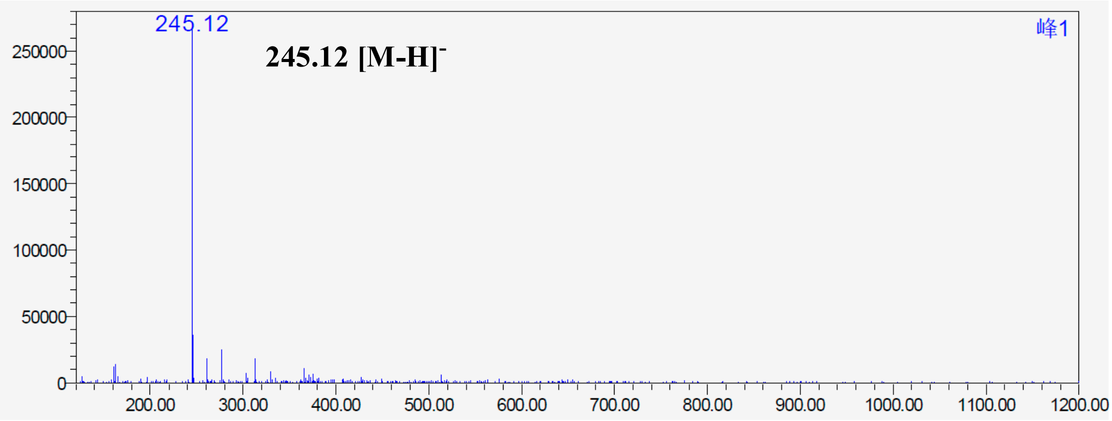

Figure 22. MS spectrum of **9.**

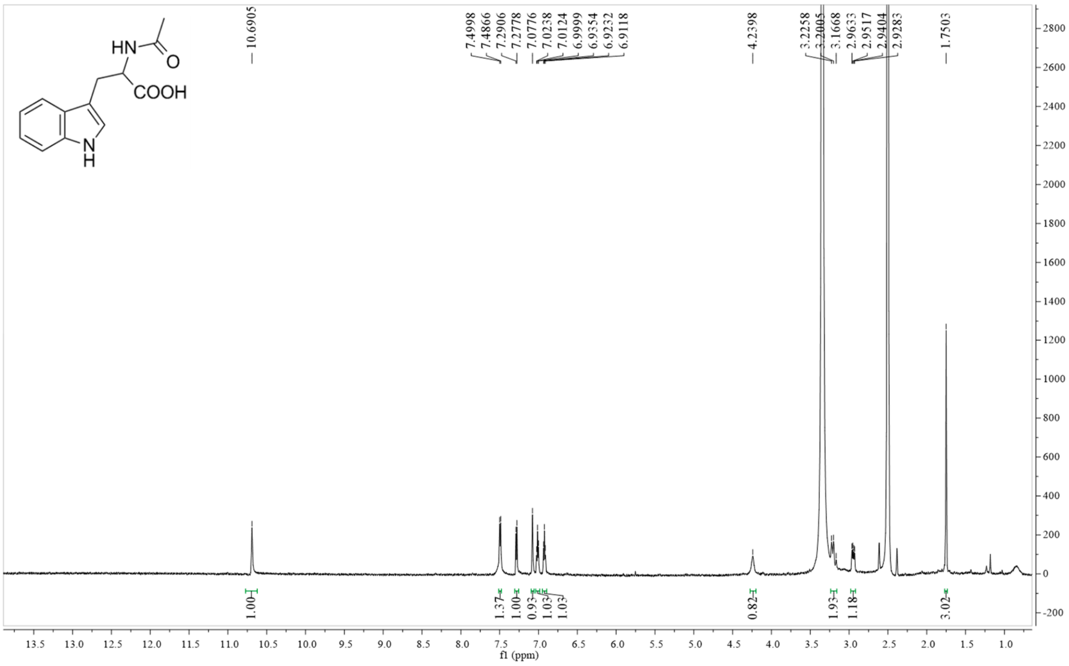

Figure 23. ^{1}H-NMR spectrum of **9.**

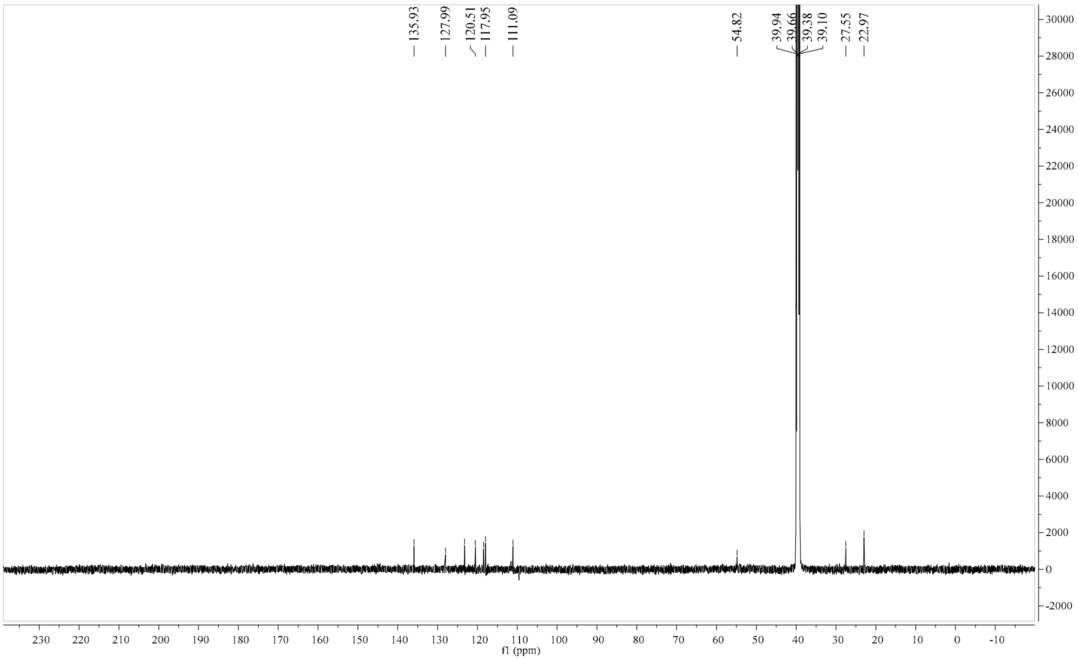

Figure 24. ^{13}C-NMR spectrum of **9.**

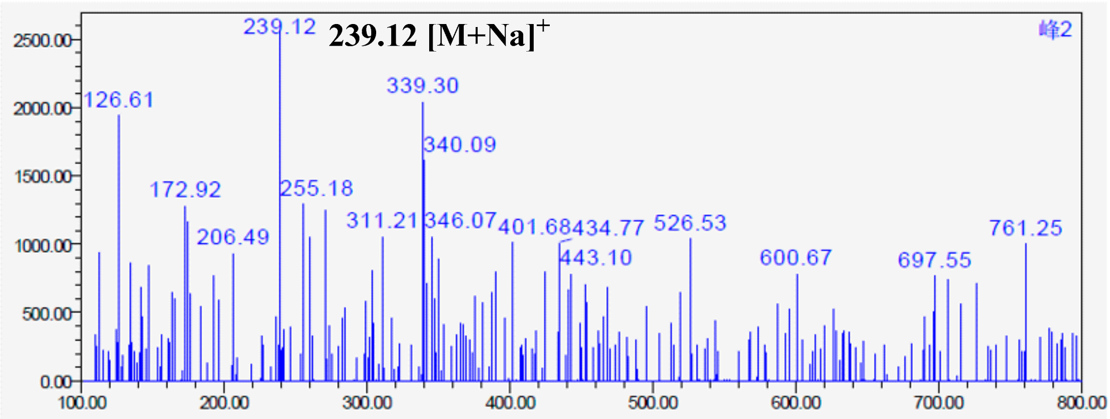

Figure 25. MS spectrum of **10.**

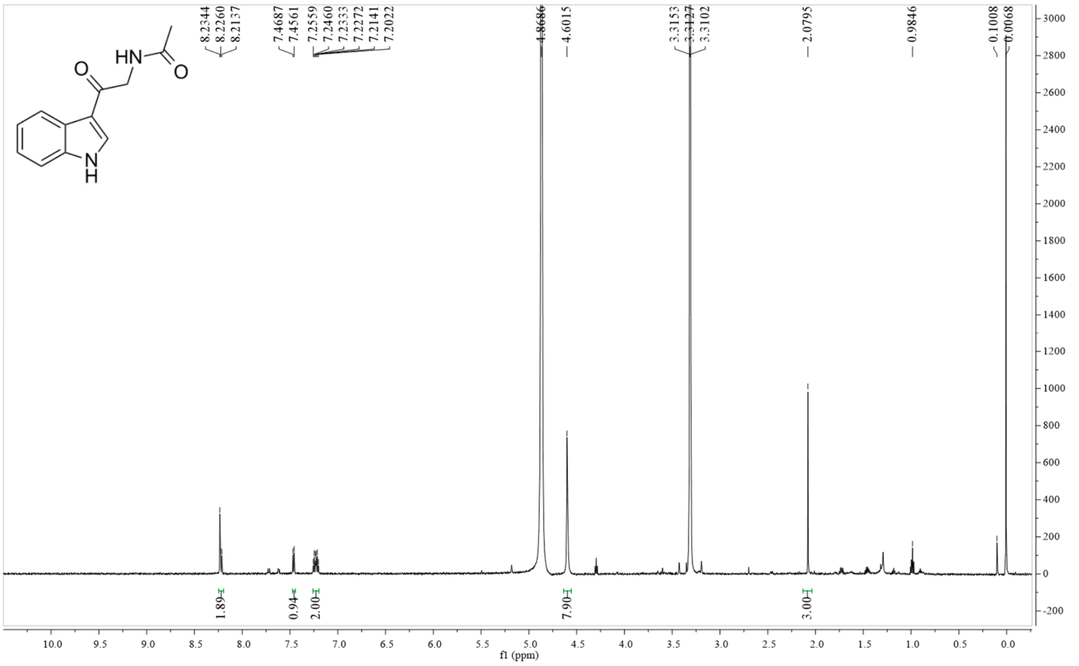

Figure 26. ^{1}H-NMR spectrum of **10.**

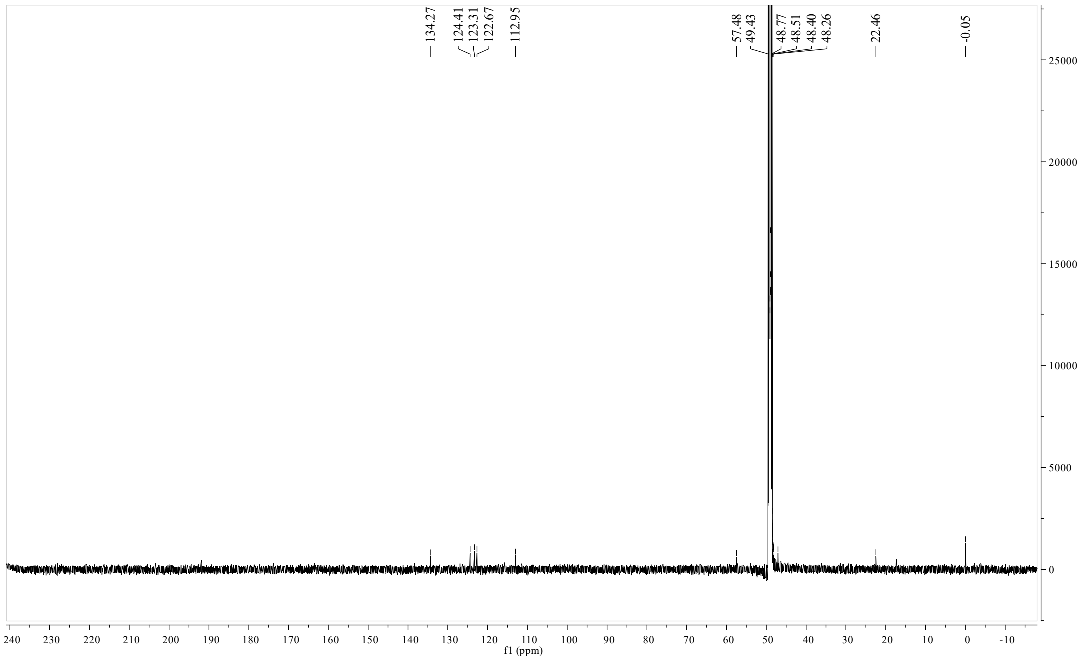

Figure 27. ^{13}C-NMR spectrum of **10**.

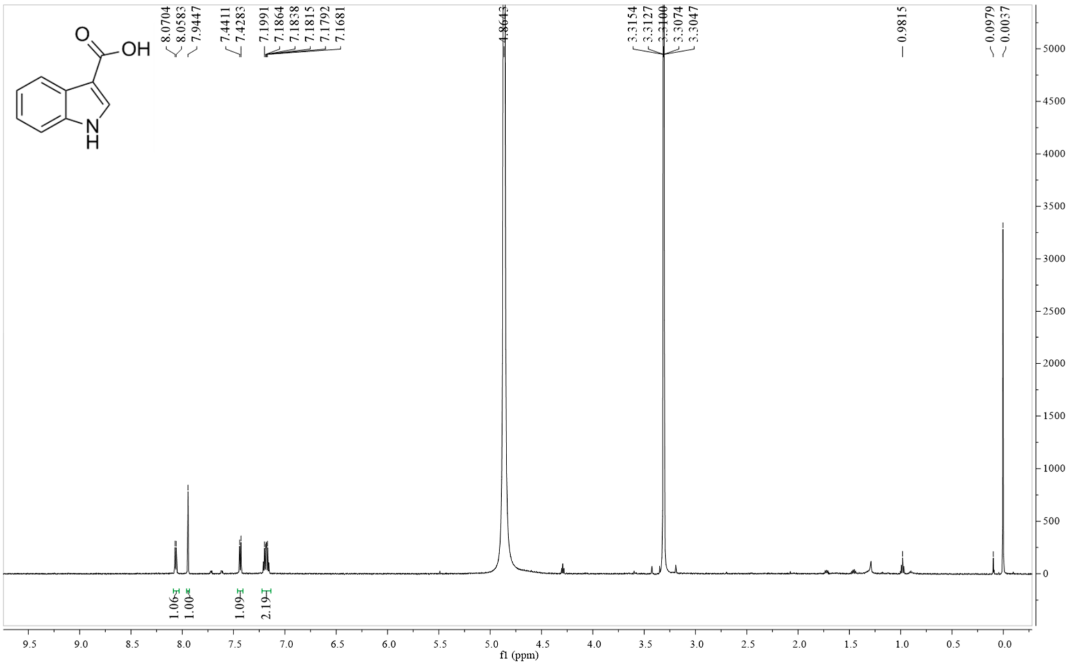

Figure 28. ^{1}H-NMR spectrum of **11.**

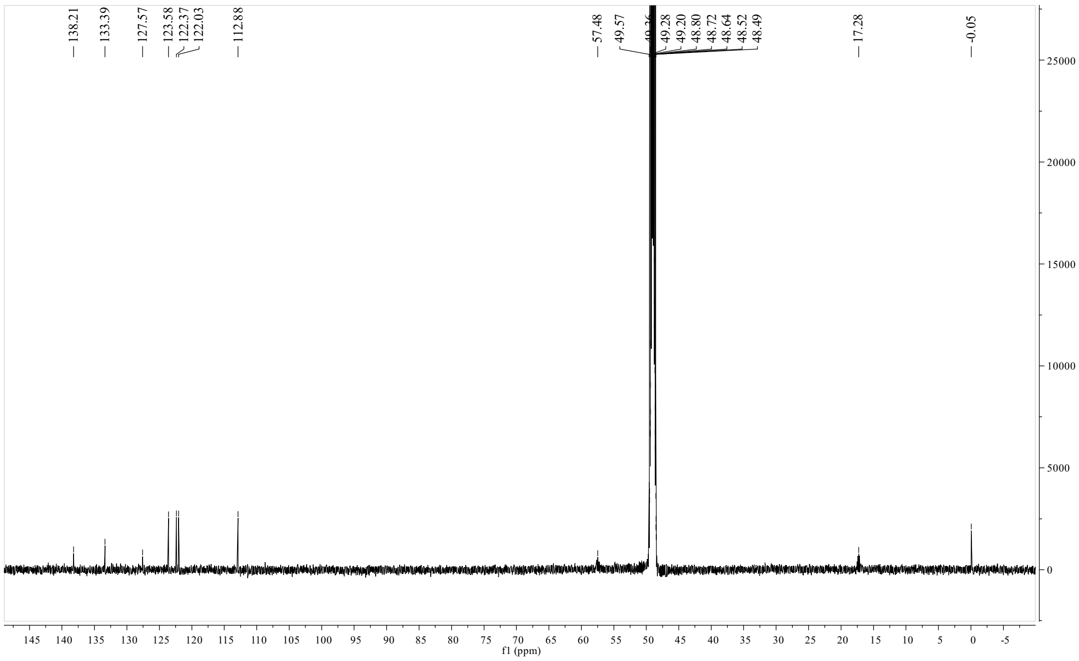

Figure 29. ^{13}C-NMR spectrum of **11.**

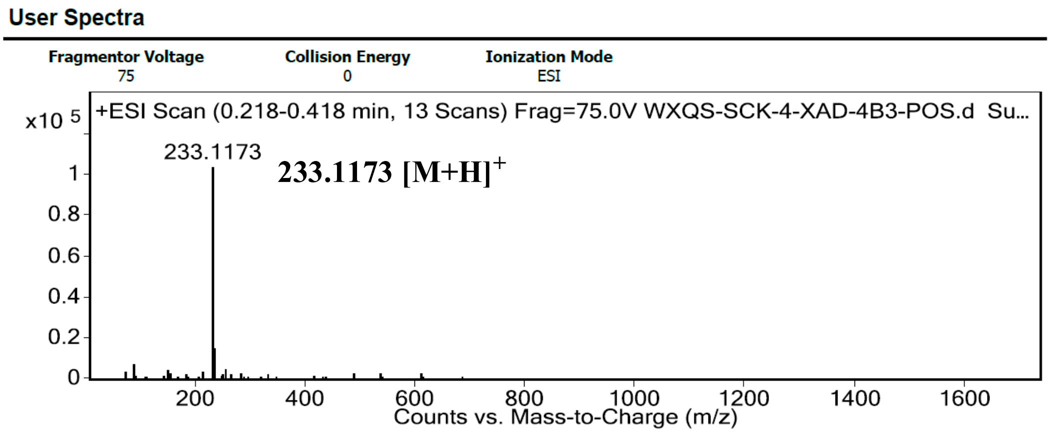

Figure 30. HR-MS spectrum of **12.**

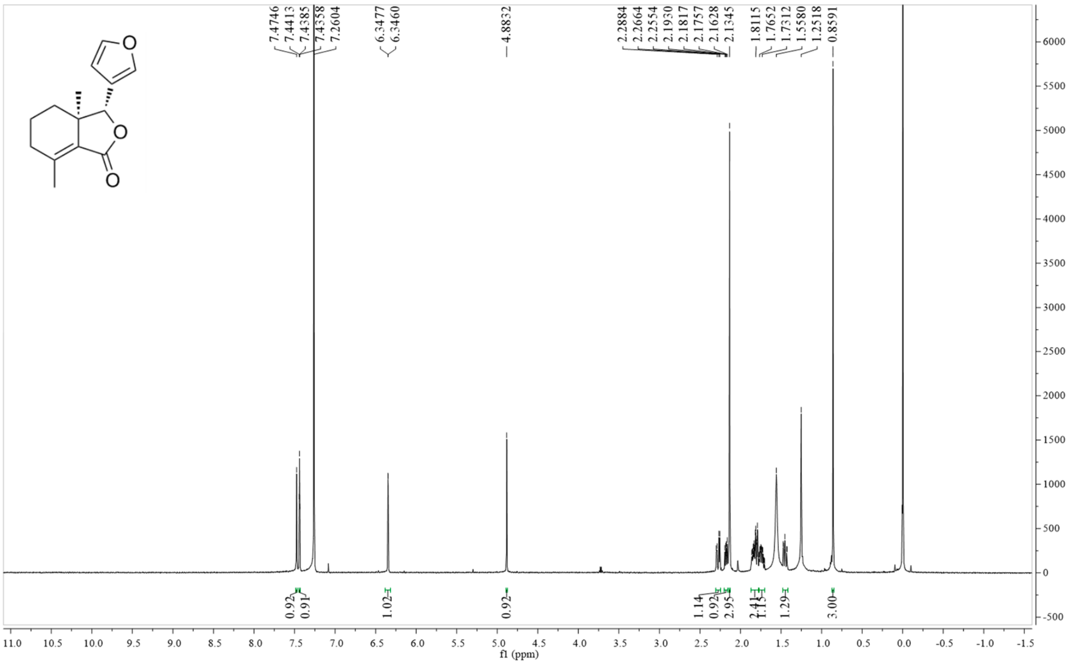

Figure 31. ^{1}H-NMR spectrum of **12.**

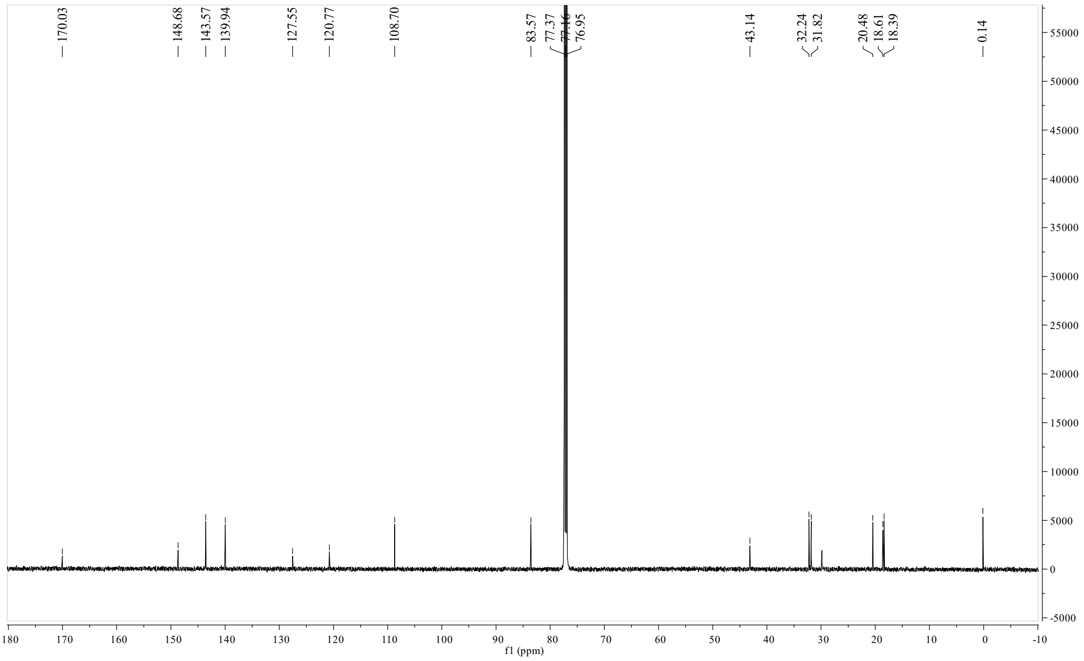

Figure 32. ^{13}C-NMR spectrum of **12.**

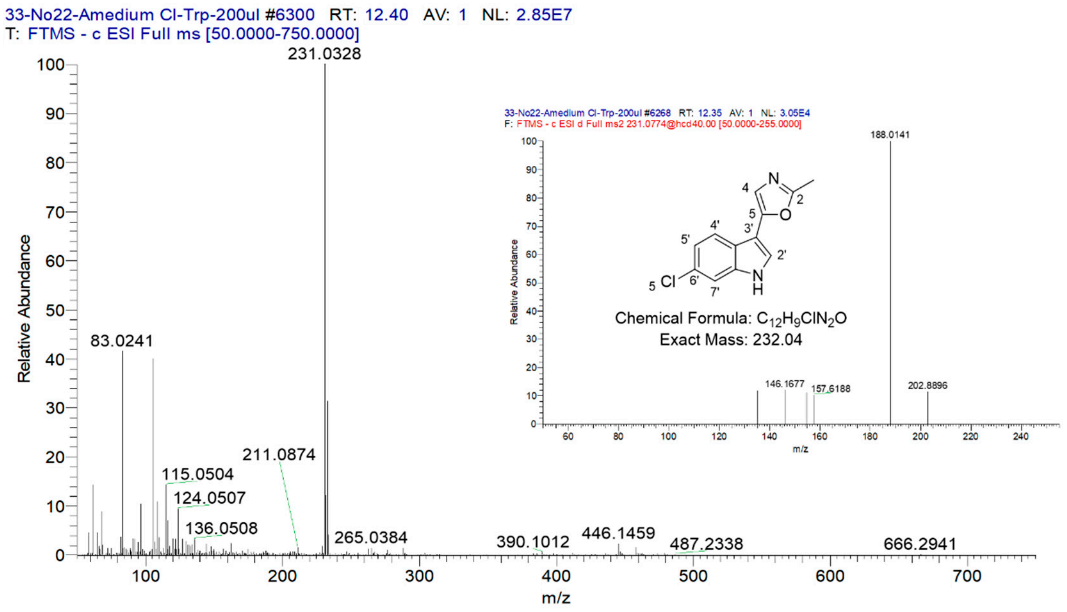

Figure 33. HR-MS spectrum of compound 6′-chloropimprinine.

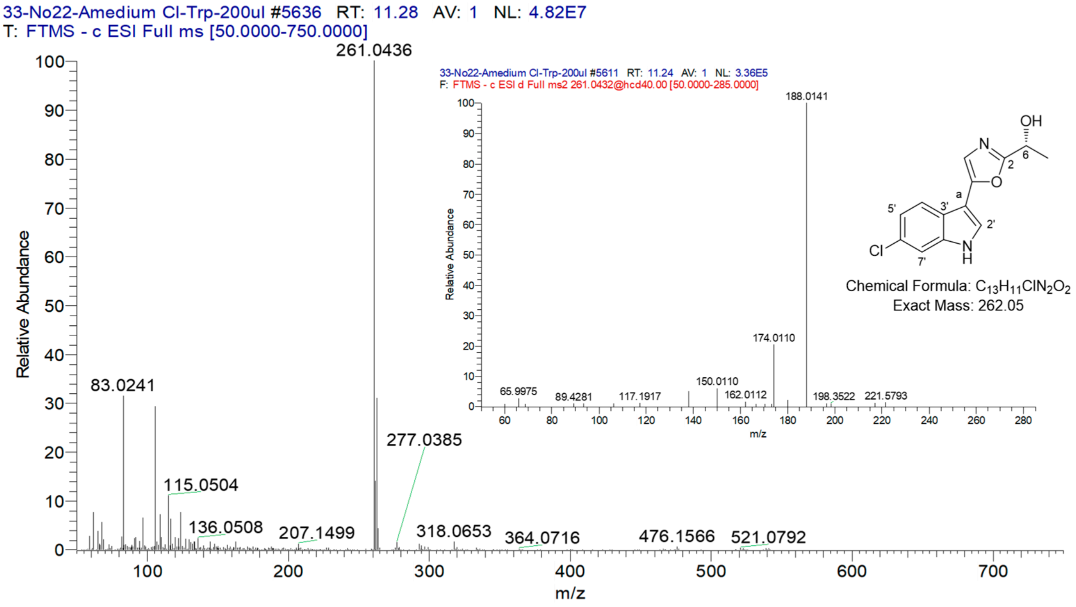

Figure 34. HR-MS spectrum of compound 6′-chloropimprinol A.

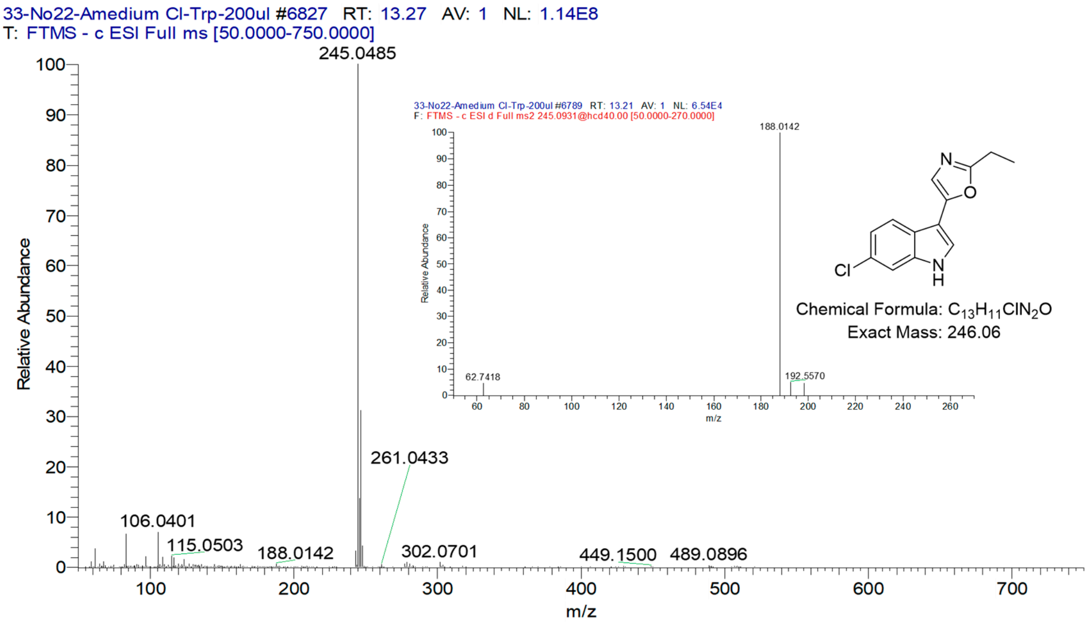

Figure 35. HR-MS spectrum of compound 6′-chloropimprinethine.

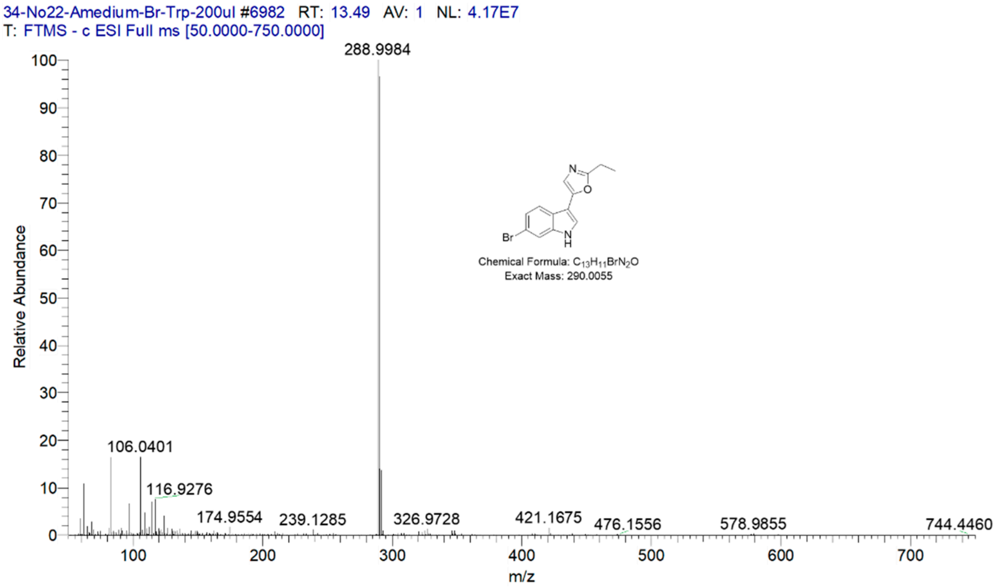

Figure 36. HR-MS spectrum of compound 6′-bromopimprinethine.

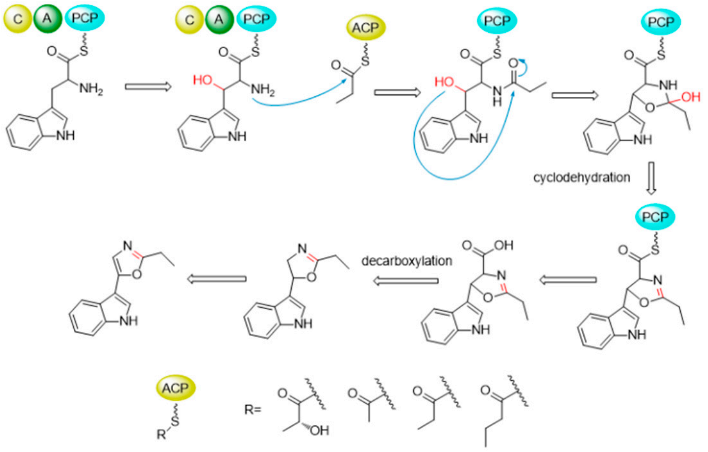

Figure 37. The possible biosynthesis pathway of pimprinine derivatives.

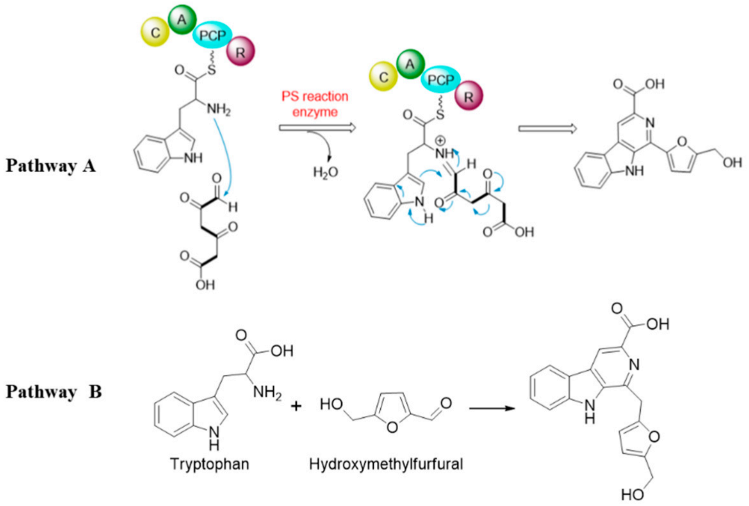

Figure 38. Two postulated pathways for the biosynthesis of β-carboline compound flazin.

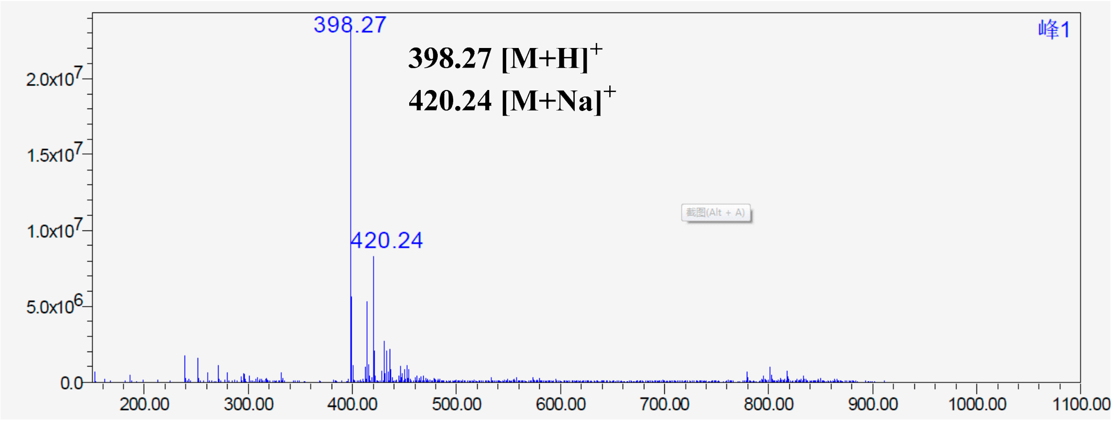

Figure 39. MS spectrum of 13.

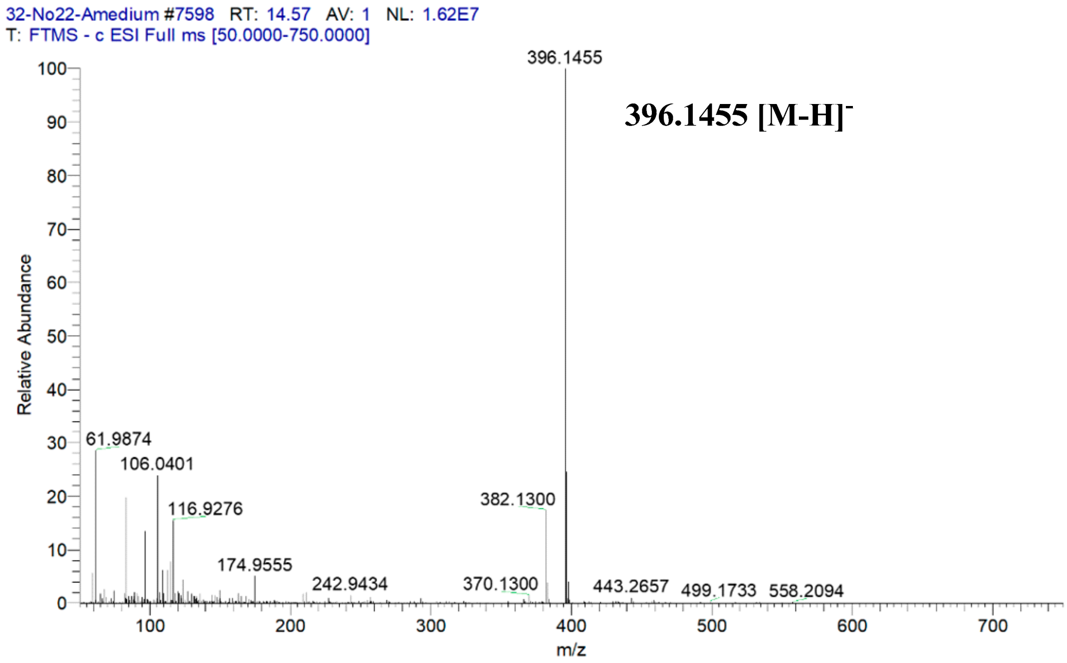

Figure 40. HR-MS spectrum of compound **13.**

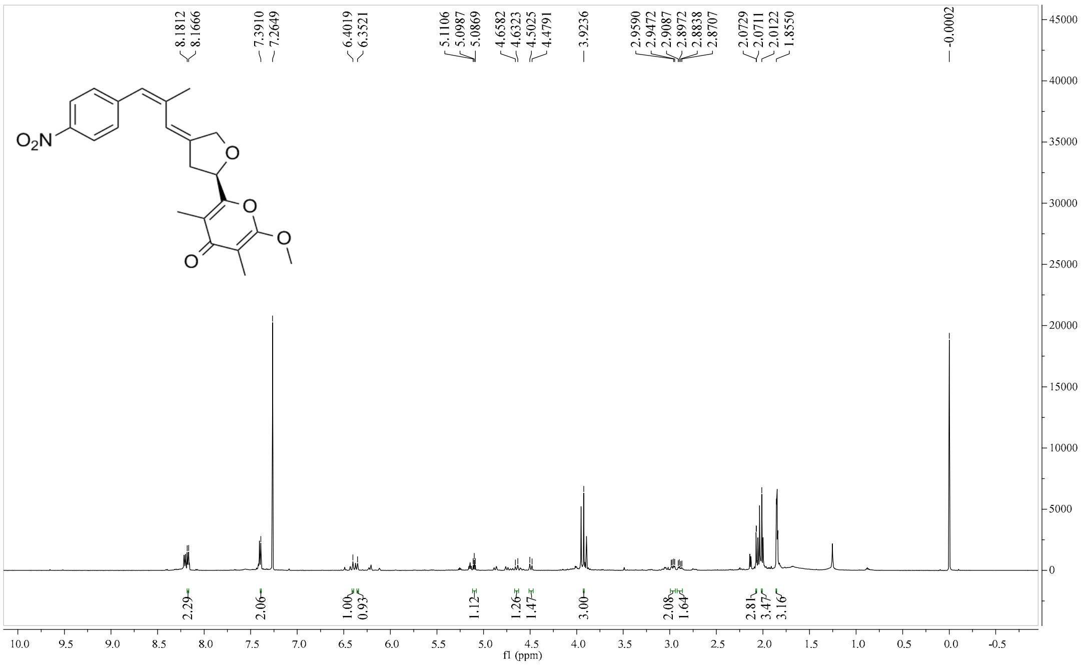

Figure 41. ^{1}H-NMR spectrum of **13.**

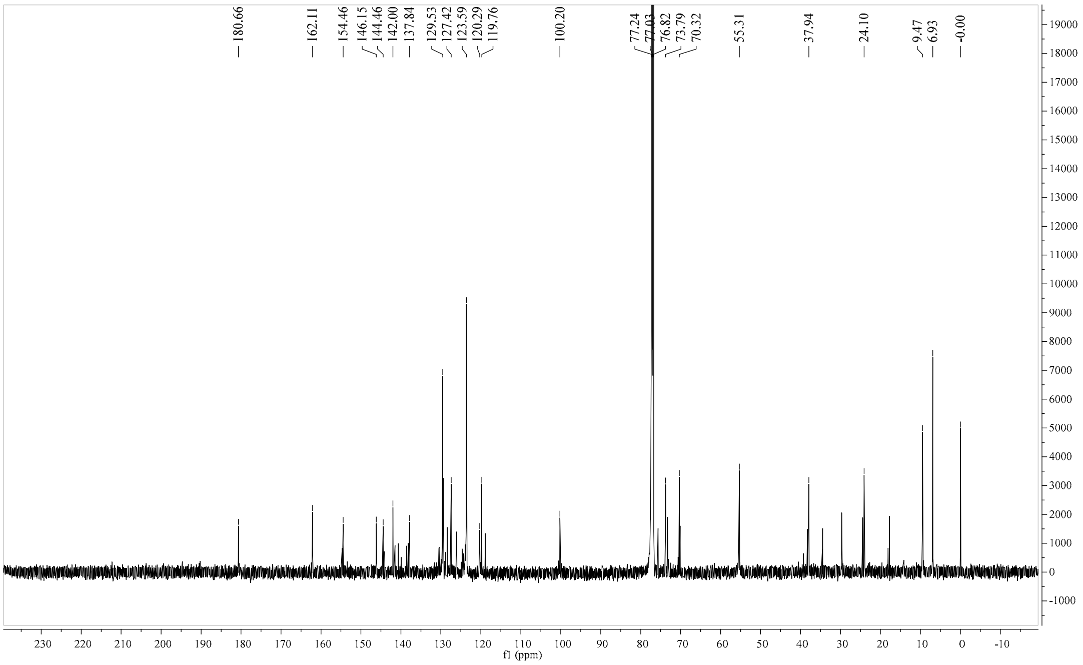

Figure 42. ^{13}C-NMR spectrum of **13.**

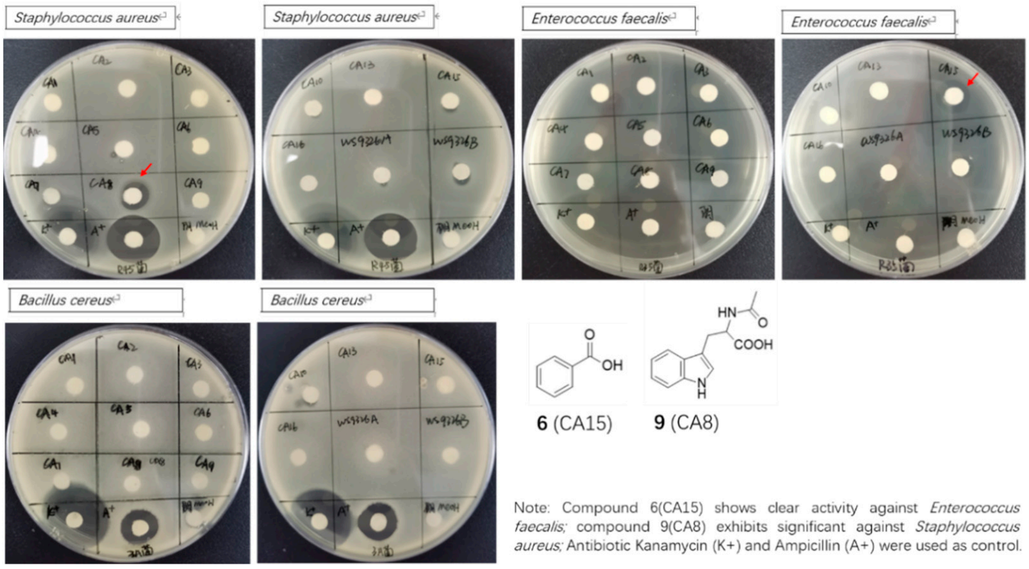

Figure 43. The antibacterial assay against *Staphylococcus aureus*, *Bacillus cereus*, and *Enterococcus faecalis*.

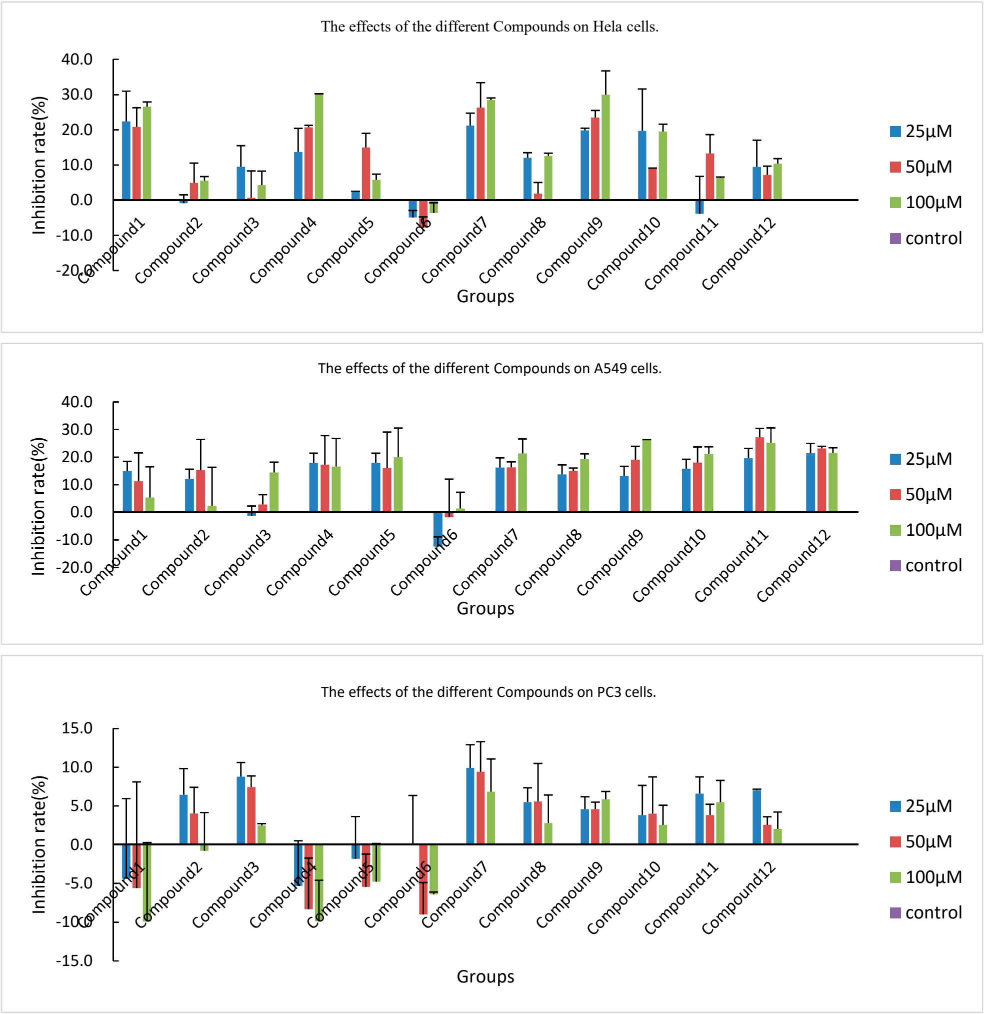

Figure 44. MTT assay to determine compound (**1-12**) cytotoxicity against hela, lung cancer (A-549), and PC-3. Three sample concentrations (25μm, 50μm and 100μm) types of cells were treated with ethanol for 48 h. The cell viability was measured by MTT assay. Values are expressed as mean and SD. *$p<0.05$ compared with the control group.

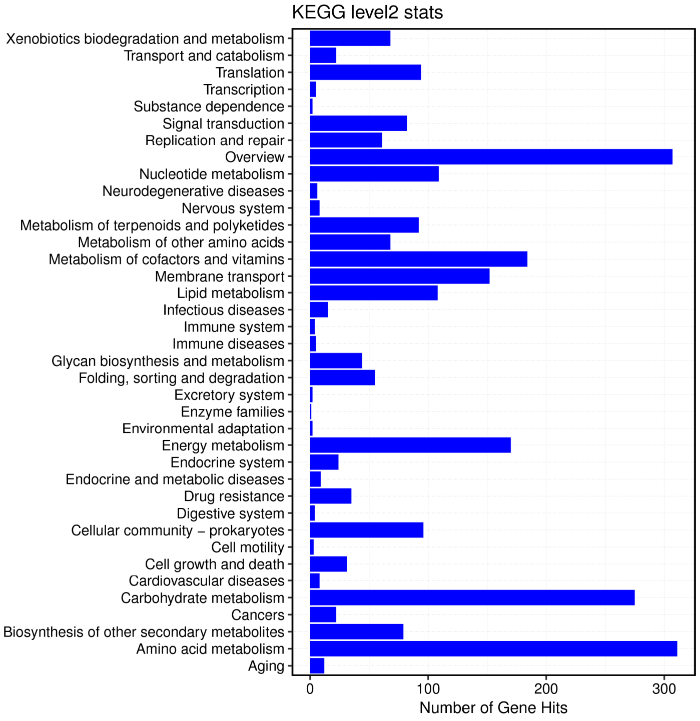

Figure 45. KEGG pathway annotation statistics of *S. netropsis* WLXQSS-4.

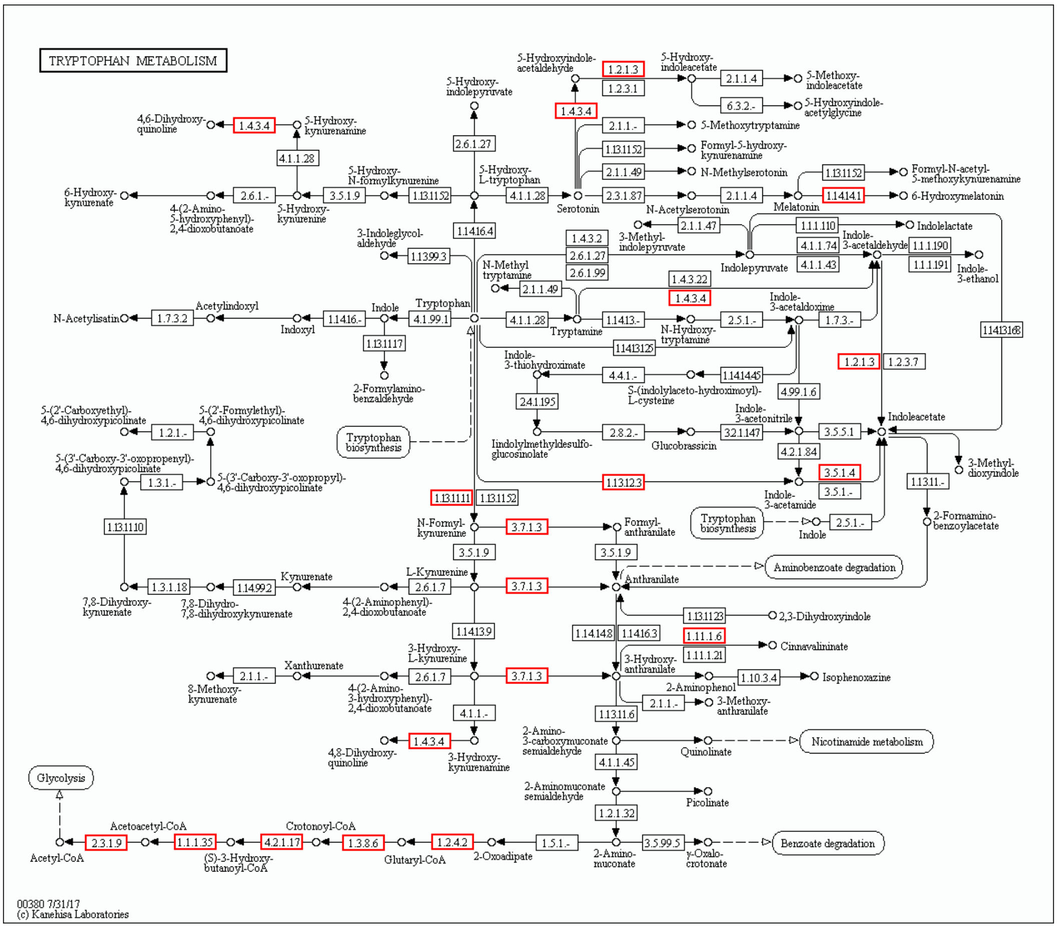

Figure 46. KEGG pathway related with the tryptophan metabolism in *S. netropsis* WLXQSS-4. The red labelled genes were found in the genome.
